# SMURF2 regulates bone homeostasis by disrupting SMAD3 interaction with vitamin D receptor in osteoblasts

**DOI:** 10.1038/ncomms14570

**Published:** 2017-02-20

**Authors:** Zhan Xu, Matthew B. Greenblatt, Guang Yan, Heng Feng, Jun Sun, Sutada Lotinun, Nicholas Brady, Roland Baron, Laurie H. Glimcher, Weiguo Zou

**Affiliations:** 1 State Key Laboratory of Cell Biology, CAS Center for Excellence in Molecular Cell Science, Institute of Biochemistry and Cell Biology, Shanghai Institutes for Biological Sciences, Chinese Academy of Sciences, Shanghai 200031, China; 2 Department of Pathology, Weill Cornell Medical College, New York, New York 10065, USA; 3 Department of Oral Medicine, Infection and Immunity, Harvard School of Dental Medicine, Boston, Massachusetts 02115, USA; 4 Department of Lab Medicine and Pathology and Masonic Cancer Center, University of Minnesota, Minneapolis, Minnesota 55455, USA; 5 Department of Medicine, Weill Cornell Medical College, New York, New York 10065, USA

## Abstract

Coordination between osteoblasts and osteoclasts is required for bone health and homeostasis. Here we show that mice deficient in SMURF2 have severe osteoporosis *in vivo.* This low bone mass phenotype is accompanied by a pronounced increase in osteoclast numbers, although *Smurf2*-deficient osteoclasts have no intrinsic alterations in activity. *Smurf2*-deficient osteoblasts display increased expression of RANKL, the central osteoclastogenic cytokine. Mechanistically, SMURF2 regulates RANKL expression by disrupting the interaction between SMAD3 and vitamin D receptor by altering SMAD3 ubiquitination. Selective deletion of *Smurf2* in the osteoblast lineage recapitulates the phenotype of germline *Smurf2*-deficient mice, indicating that SMURF2 regulates osteoblast-dependent osteoclast activity rather than directly affecting the osteoclast. Our results reveal SMURF2 as an important regulator of the critical communication between osteoblasts and osteoclasts. Furthermore, the bone mass phenotype in *Smurf2*- and *Smurf1*-deficient mice is opposite, indicating that SMURF2 has a non-overlapping and, in some respects, opposite function to SMURF1.

## Abstract (web summary)

The balance between osteoclast and osteoblast-mediated bone turnover is essential for bone health and homeostasis. Here the authors show that both germline and osteoblast-specific *Smurf2*-deficient mice have osteoporosis as a result of increased osteoblast RANKL production and excess osteoclastogenesis.

Bone remodelling is an important physiological process to preserve the structural integrity of the skeleton, and is achieved through the balance between bone formation by osteoblasts and bone resorption by osteoclasts^1^,^2^. Osteoblasts, which originate from mesenchymal precursors, secrete the extracellular matrix required for bone mineralization during both embryonic development and the postnatal remodelling that is critical for bone homeostasis in adults^1^. Osteoclasts, which are multinucleated giant cells originating from the myelomonocytic lineage, degrade existing bone through an acidic and enzymatic process^2^. Imbalance between the activity of osteoblasts and osteoclasts during bone remodelling can result in reduced bone mass, which underlies the pathogenesis of osteoporosis, the most common skeletal disease^3^.

To ensure balance between the catabolic and anabolic activities during skeletal remodelling, many factors mediate the coordination between osteoblasts and osteoclasts. For example, during bone resorption, active transforming growth factor-β1 (TGF-β1) is released from bone matrix, recruiting bone marrow (BM) mesenchymal stem cells, which can be further differentiated to osteoblasts^4^,^5^. RANKL secreted by osteoblast lineage cells, including both osteoblasts and osteocytes, binds to its receptor RANK on the cell membrane of osteoclast progenitors, promoting osteoclast formation and function^2^,^6^,^7^. The expression of *RANKL* is regulated at the cellular level by multiple signalling pathways, including schnurri-3 (ref. 8), EBF2 (ref. 9), parathyroid hormone (PTH), PTH-related protein, TGF-β and vitamin D^10^,^11^,^12^,^13^.

Nedd4 E3 ligases are members of the HECT E3 ubiquitin ligase family and regulate ubiquitination-mediated protein degradation^14^. Among them, Smad regulatory factors (SMURFs) are the subject of much interest due to their modulation of TGF-β and BMP signalling. Vertebrates have two highly related Smurf genes: *Smurf1* and *Smurf2*, which have similar expression patterns, demonstrated by *in situ* hybridization analysis, and have partially redundant functions^15^. Consistent with this notion, *Smurf1*^*−/−*^; *Smurf2*^*−/−*^ (Smurf DKO) mice display embryonic lethality at around E12.5. However, single *Smurf1*^*−/−*^ or *Smurf2*^*−/−*^ mice have no overt defects in embryogenesis^16^. Both SMURF1 and SMURF2 are expressed by osteoblasts and proliferating chondrocytes, but not by osteoclasts^15^. SMURF1 regulates osteoblast differentiation by promoting the degradation of MEKK2 and *Smurf1*^*−/−*^ mice display increased bone mass^15^,^17^. *Smurf2* transcript levels are increased in the tibias of *Smurf1*^*−/−*^ mice, suggesting that SMURF2 may partially compensate for the loss of SMURF1 during osteoblast differentiation and function^15^ and raising the possibility that SMURF2 might also be a repressor of bone formation.

Here we evaluate the contribution of the Nedd4 E3 ligase SMURF2 to osteoblast activity by examining the skeletal phenotype of *Smurf2*^*−/−*^ mice. Surprisingly, mice lacking *Smurf2* have low bone mass, the opposite to the phenotype of *Smurf1*-deficient mice. *Smurf2*^*−/−*^ mice have an increase in osteoclast numbers and enhanced bone resorption. *Smurf2*^*−/−*^ osteoblasts have increased expression of *RANKL* due to alterations in SMURF2-mediated SMAD3 ubiquitination. Our results identify SMURF2 as a regulator between osteoblasts and osteoclasts, the disruption of which results in low bone mass. Furthermore, this study shows that SMURF2 has a non-overlapping and, in some respects, an opposite function to SMURF1.

## Results

### *Smurf2* deficiency increases osteoblast differentiation

**Figure 1:**
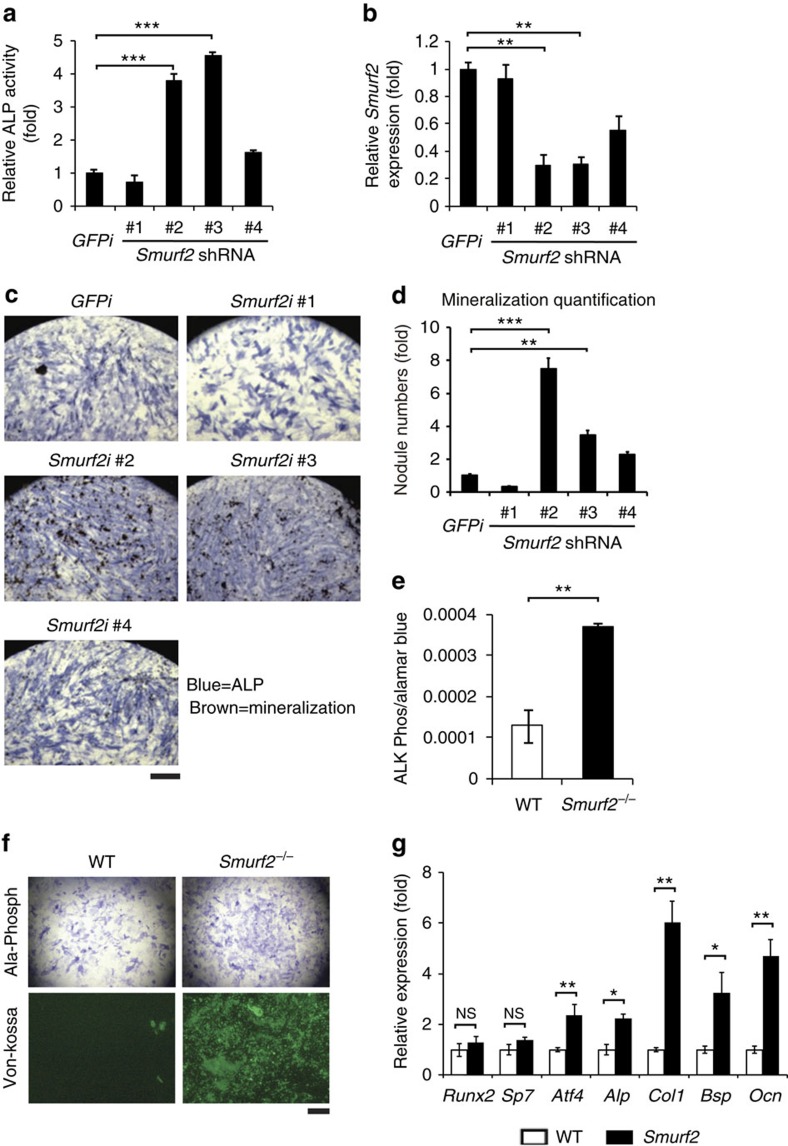
Osteoblast differentiation increased in *Smurf2* knockdown and knockout cells. (**a**,**b**) Analysis of ALP and *Smurf2* expression in human mesenchymal stem cells (hMSCs) infected with lentivirus-expressing GFP control or *Smurf2* shRNAs. (**c**) Von Kossa staining representative images for combined ALP (blue) and Von Kossa (brown) staining of hMSCs infected with lentivirus-expressing control or *Smurf2* shRNAs cultured in osteoblast differentiation medium for 21 days. Scale bar, 0.1 cm. (**d**) Bone nodule mineralization of hMSCs determined by Von Kossa staining. The bar graph displays the quantification of Von Kossa-positive mineralized nodules. (**e**,**f**) Analysis of ALP expression and Von Kossa staining of primary osteoblasts cultured in osteoblast differentiation medium for 7 and 21 days. Scale bar, 0.1 cm. (**g**) Quantitative reverse transcriptase–PCR analysis of osteoblast genes in primary osteoblasts from neonatal 5-day-old mice cultured in osteoblast differentiation medium for 7 days. Data represent mean±s.d. (*n*=10 for each genotype, *P<0.05, **P<0.01, ***P<0.001, NS, not significant. Student's *t*-test).

We previously performed an RNA interference-based loss-of-function screen in human mesenchymal stem cells to identify novel regulators of osteoblast differentiation^18^. We specifically screened nine members of the Nedd4 E3 ligase family, using robust induction of alkaline phosphatase (ALP), an early marker of osteoblast differentiation, as readout (Supplementary Fig. 1). Short hairpin RNAs (shRNAs) directed against the Nedd4 E3 ligase *Smurf2* reduced *Smurf2* expression and increased ALP level (Fig. 1a,b; Supplementary Fig. 1). Consistently, *Smurf2*-targeted shRNAs also induced elevated levels of mineral deposition as determined by Von Kossa staining (Fig. 1c,d). To further explore the function of SMURF2 during osteoblastogenesis, we investigated the function of SMURF2 in primary osteoblasts. Mice lacking *Smurf2* generated by deletion of exon 9 were previously described to be viable and fertile^16^. Loss of SMURF2 was confirmed by immunoblotting extracts of the lung and liver (Supplementary Fig. 2A). *In vitro*, primary *Smurf2*^*−/−*^ calvarial osteoblasts displayed an increased induction of ALP and enhanced mineralized matrix formation, confirming the results of the shRNA screen (Fig. 1e,f). Consistent with this enhanced differentiation, expression of characteristic osteoblast markers including tissue-nonspecific *Alp*, collegen1 alpha 1 (*Col1a1*), bone sialoprotein (*Bsp*), osteocalcin (*Ocn*) and *Atf4* were increased in *Smurf2*^*−/−*^ osteoblasts (Fig. 1g). Collectively, these data demonstrate that knockdown of *Smurf2* in human mesenchymal stem cells and knockout of *Smurf2* in primary osteoblasts augment osteoblast differentiation *in vitro*.

### *Smurf2* ^*−/−*^ mice show a reduced bone mass phenotype

**Figure 2:**
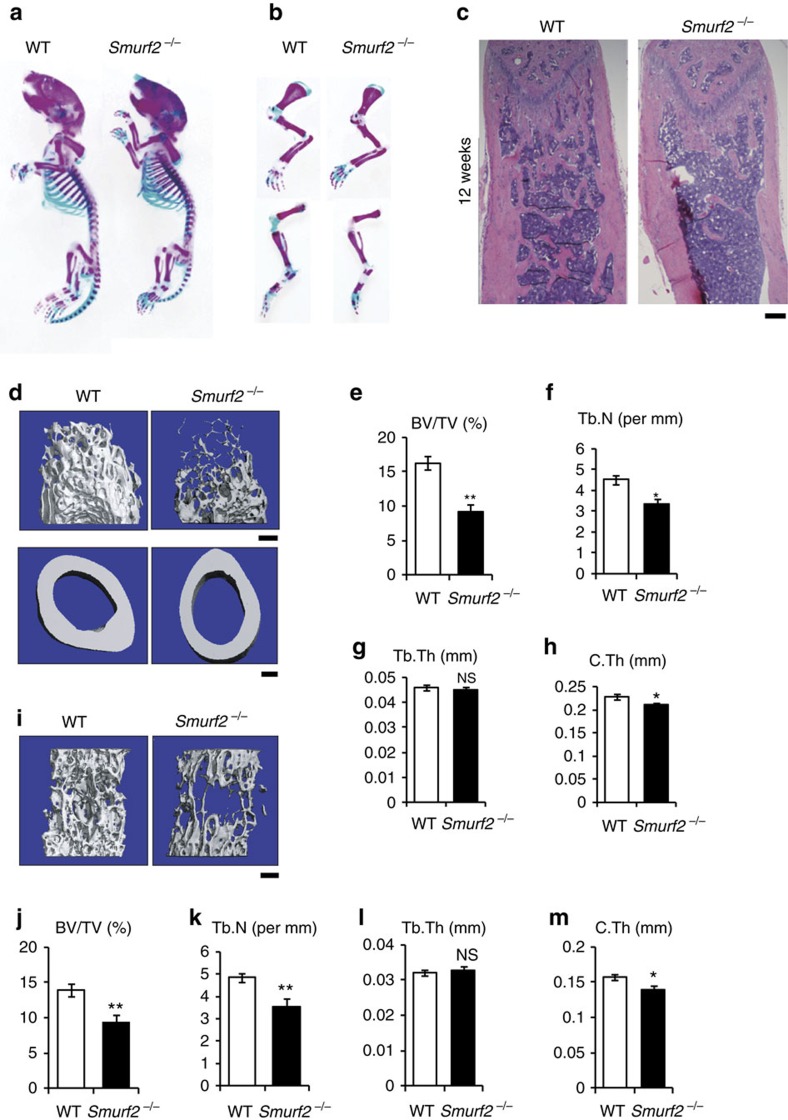
*Smurf2*^*−/−*^ mice have a reduced bone mass phenotype. (**a**) Alcian blue/alizarin red staining of the whole skeleton from 1-week-old WT and *Smurf2*^*−/−*^ male littermates. (**b**) Alcian blue/alizarin red staining of forelimb and hindlimb from 1-week-old WT and *Smurf2*^*−/−*^ male littermates. (**c**) Haematoxylin and eosin staining of distal femur of 12-week-old male WT and *Smurf2*^*−/−*^ mice. Scale bar, 500 μm. (**d**–**h**) μ-QCT analysis of distal femurs from 12-week-old male WT and *Smurf2*^*−/−*^ mice for bone volume per tissue volume (BV/TV), trabecular number (Tb.N), trabecular thickness (Tb.Th) and cortical thickness (C.Th). Scale bar, 500 μm. (**i**–**m**) μ-QCT analysis of the fifth lumbar vertebra from 12-week-old male WT and *Smurf2*^*−/−*^ mice for BV/TV, Tb.N, Tb.Th and C.Th. Scale bar, 500 μm. Data represent mean±s.d. (*n*=6 for each genotype, **P<0.01, *P<0.05, NS, not significant. Student's *t*-test).

Nevertheless, we have noted that many genes identified in *in vitro* shRNA silencing screens to alter osteoblast differentiation do not do so *in vivo* (unpublished data). To determine the *in vivo* relevance of SMURF2 within the skeletal system, we examined the skeleton of 1-week-old *Smurf2*^*−/−*^ mice by alcian blue and alizarin red staining. *Smurf2*^*−/−*^ mice had no overt developmental defects at weaning, and *Smurf2*^*−/−*^ mice were smaller than control littermates in terms of both body size and weight (Fig. 2a,b; Supplementary Fig. 2B). *Smurf2*^*−/−*^ mice displayed normal growth plate architecture. However, less trabecular bone was present in femurs of 12-week-old *Smurf2*^*−/−*^ mice, compared to wild-type (WT) controls (Fig. 2c). Further analysis by micro quantitative computed tomography (μ-QCT) demonstrated that the distal femurs of *Smurf2*^*−/−*^ mice had decreased trabecular bone volume (BV/TV), trabecular number (Tb.N) and cortical thickness (C.Th) with normal trabecular thickness (Tb.Th; Fig. 2d–h). The fifth lumbar vertebra of *Smurf2*^*−/−*^ mice similarly showed reduced BV/TV, Tb.N and C.Th, but not Tb.Th compared with WT littermates (Fig. 2i–m). Thus, in contrast to the increased bone density in *Smurf1*-deficient mice, loss of *Smurf2* in mice resulted in reduced bone mass. As seen in older mice, *Smurf2*^*−/−*^ mice begin to show low bone mass even at 1 week of age (Supplementary Fig. 2C). *In situ* hybridization for Col1 expression was also performed. As shown in Supplementary Fig. 2D, the extent of *Col1* expression was reduced in *Smurf2*^*−/−*^ mice, though there did appear to be some increase in the per-cell intensity of staining within this reduced area. Thus, SMURF2 plays a similar role in regulating bone mass during both the early modelling and later remodelling stages of bone formation.

### *Smurf2* ^*−/−*^ mice have increased bone resorption

**Figure 3:**
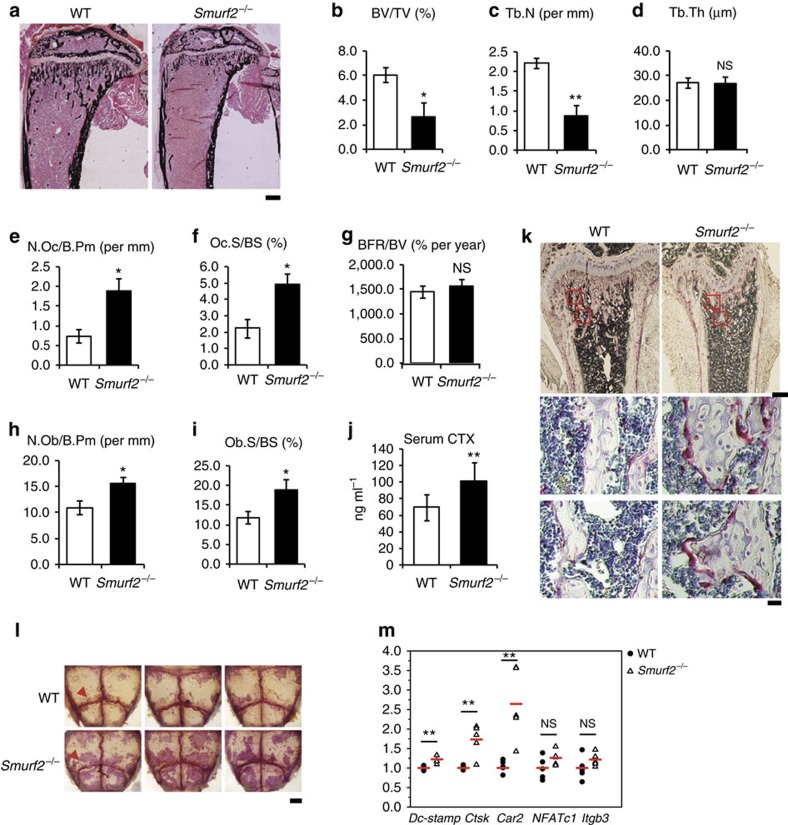
*Smurf2*^*−/−*^ mice have increased bone resorption. (**a**–**i**) Histomorphometric analysis of proximal tibia from 5-week-old WT and *Smurf2*^*−/−*^ male mice for bone volume per tissue volume (BV/TV), trabecular number (Tb.N), trabecular thickness (Tb.Ths), number of osteoclasts per bone perimeter (N.Oc/B.Pm), osteoclast surface per bone surface (Oc.S/BS), bone formation rate per bone volume (BFR/BV), number of osteoblasts per bone perimeter (N.Ob/B.Pm) and osteoblast surface per bone surface (Ob.S/BS). Scale bar, 500 μm. Data represent mean±s.d. (*n*=6 for each genotype, **P<0.01, *P<0.05, NS, not significant. Student's *t*-test). (**j**) Serum CTX levels in 5-week-old male WT and *Smurf2*^*−/−*^ animals. (**k**) Histological sections of 4-week-old male mice's control and *Smurf2*^*−/−*^ tibias stained for tartrate-resistant acid phosphatase (TRAP). Scale bars, upper 500 μm; bottom 10 μm. (**l**) Representative photographs of whole-mount TRAP staining of 4-week-old male mice (pink, arrows highlight prominent staining near sutures). Scale bar, 1 mm. (**m**) Quantitative reverse transcriptase–PCR analysis of osteoclast-specific genes in calvarium of 4-week-old male mice. Data represent mean±s.d. (*n*=5 for each genotype, ***P<0.001, **P<0.01, *P<0.05, NS, not significant. Student's *t*-test).

To understand the relative contributions of osteoblast and osteoclast activities to the osteopenia seen in *Smurf2*^*−/−*^ mice, we next performed histomorphometric analysis of WT and *Smurf2*^*−/−*^ mice to evaluate static and dynamic parameters of bone formation and resorption (Fig. 3a–i; Supplementary Table 1). Consistent with the μ-QCT data, histomorphometry also showed that *Smurf2*^*−/−*^mice had a significant decrease in both BV/TV and Tb.N with a concomitant increase in trabecular spacing (281%), but no change in Tb.Th (Fig. 3b–d; Supplementary Table 1), a characteristic phenotype associated with increased bone resorption. Indeed, the decreased bone volume was associated with a significant increase in both the number and bone surface covered by osteoclasts (N.Oc/Bpm and Oc.S/BS; Fig. 3e,f). In addition, the numbers of osteoblasts per bone perimeter (N.Ob/B.Pm) and osteoblast surface per bone surface (Ob.S/BS) were increased in *Smurf2*^*−/−*^mice compared to the WT control mice. However, the bone formation rate normalized to bone volume was not significantly increased (Fig. 3g–i; Supplementary Table 1). Given that the unexpected decrease in bone volume in *Smurf2*^*−/−*^mice was accompanied by increases in osteoclast number, we next examined levels of a serum marker of bone resorption activity, collagen type 1 cross-linked C-telopeptide (CTX). As shown in Fig. 3j, CTX was significantly increased in *Smurf2*^*−/−*^ mice, indicating the elevated bone resorption activity in *Smurf2*^*−/−*^ mice. We next performed a more extensive histochemical analysis of osteoclasts and observed quantitative increases in osteoclast numbers along the surface of trabecular bone (Fig. 3k) and in whole-mount skull preparations (Fig. 3l), indicated by the presence of tartrate-resistant acid phosphatase-positive (TRAP-positive) osteoclasts. Consistent with the increase of osteoclast numbers, expression of characteristic osteoclast marker genes including *Dc-stamp*, cathepsin K (*Ctsk*) and carbonic anhydrase 2 (*Car2*) were increased in the calvaria of *Smurf2*^*−/−*^ mice (Fig. 3m), although the expression level of *NFATc1* and integrin beta 3 (*Itgb3*) was not much affected in *Smurf2-*deficient mice, which could be due to the expression of *NFATc1* and *Itgb3* in other cell types besides osteoclasts. Collectively, these data indicate that the decrease in bone volume in *Smurf2*^*−/−*^ mice is due to increased bone resorption.

### *Smurf2* ^*−/−*^ osteoblasts have increased RANKL expression

**Figure 4:**
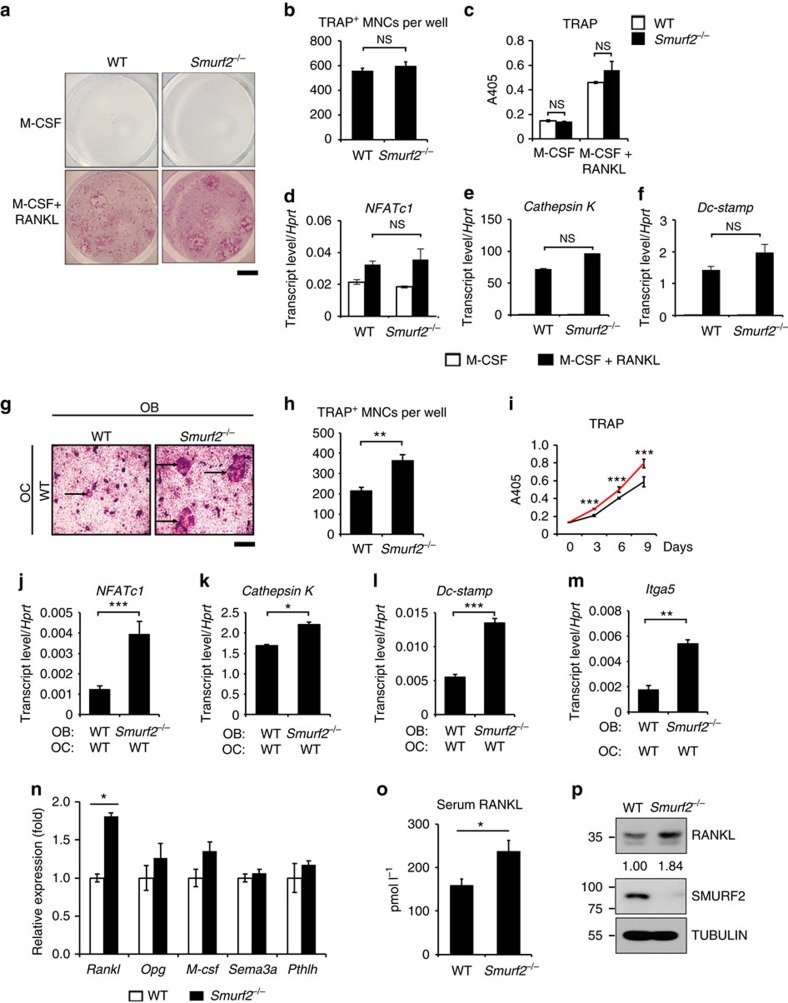
*Smurf2*^*−/−*^ osteoblasts express high levels of RANKL driving osteoclastogenesis. (**a**–**f**) Osteoclastogenesis of bone marrow cells from 4-week-old mice *in vitro*. (**a**) TRAP staining of osteoclasts in culture. Scale bar, 0.5 cm. (**b**) Statistical analysis of TRAP-positive cell numbers in the osteoclast differentiation culture. (**c**) Culture supernatants were assayed for TRAP activity via colorimetric readout (A405). (**d**–**f**) Gene expression analysis of osteoclast cultures was examined by quantitative reverse transcriptase–PCR. (**g**–**m**) Osteoclastogenesis of osteoblasts/bone marrow cells by OB-OC co-culture *in vitro* using neonatal calvarial osteoblasts. (**g**) TRAP staining of osteoclasts in co-culture, Scale bar, 0.5 mm. (**h**) Statistical analysis of TRAP-positive cell numbers in the co-culture shown in **g**. (**i**) Co-culture supernatants were assayed for TRAP activity via colorimetric readout (A405). Red line, osteoblast cells were from *Smurf2*^*−/−*^ mice; black line, osteoblast cells were from WT mice. (**j**–**m**) Gene expression analysis of osteoblasts/bone marrow cells co-cultures was examined by quantitative reverse transcriptase–PCR. (**n**) Gene expression of major osteoclast differentiation regulators in neonatal calvarial osteoblasts. (**o**) Serum RANKL was determined from 5-week-old male WT and *Smurf2*^*−/−*^ mice. Data represent mean±s.d. (*n*=10 for each genotype, ***P<0.001, **P<0.01, *P<0.05, NS, not significant. Student's *t*-test). (**p**) Expression of RANKL, SMURF2 and TUBULIN in primary neonatal calvarial.

To address whether the apparent increase in osteoclast activity seen in *Smurf2*^*−/−*^ mice was intrinsic to the osteoclast lineage, BM from *Smurf2*^*−/−*^ mice and WT littermates was collected and cultured in the presence of the osteoclastogenic factors RANKL and macrophage colony-stimulating factor (M-CSF). Osteoclast differentiation was normal in *Smurf2*^*−/−*^ BM macrophages (BMMs), as indicated by a similar number of multinucleated TRAP-positive osteoclasts and supernatant TRAP activity, when compared to BMMs from WT mice (Fig. 4a–c). Further, induction of makers of osteoclast differentiation, including *NFATc1*, *Cathepsin K* and *Dc-stamp*, was comparable in WT and *Smurf2*^*−/−*^ osteoclasts (Fig. 4d–f). Thus, osteoclast differentiation is intact in the absence of *Smurf2*. These results suggest that the elevated bone resorption observed in *Smurf2*^*−/−*^ mice is extrinsic to the osteoclast lineage, which is also consistent with the very low level of expression of *Smurf2* in osteoclasts.

Osteoblastic lineage cells support osteoclast differentiation *in vitro* and *in vivo* by expressing RANKL, a major inducer of osteoclast differentiation and function^2^,^6^,^7^,^19^. To investigate the ability of *Smurf2*^*−/−*^ osteoblast lineage cells to support osteoclastogenesis, we performed co-culture experiments. Osteoblasts from *Smurf2*^*−/−*^ mice showed an enhanced ability to support osteoclastogenesis, as indicated by the number of giant multinucleated cells and the TRAP activity of culture supernatants (Fig. 4g–i). Similarly, RNA obtained from osteoclasts cultured with *Smurf2*^*−/−*^ osteoblasts displayed increased expression of terminal markers of osteoclast differentiation, including *NFATc1*, *Cathepsin K*, *Dc-stamp* and *Itga5* (Fig. 4j–m). These results demonstrate that SMURF2 acts within osteoblasts to regulate osteoclast differentiation, prompting us to identify the osteoclastogenic factor(s) produced by osteoblasts that are regulated by SMURF2. RANKL and OPG, which are expressed in osteoblast cells, are major regulators of osteoclast differentiation^2^,^4^,^20^. Indeed, *Smurf2*^*−/−*^ calvarial osteoblasts had increased *RANKL* expression but there was no effect on *OPG* expression (Fig. 4n). The expression of other osteoclast differentiation regulators, including *M-CSF*, Semaphorin 3A (*Sema3a*)^21^ and parathyroid hormone-like peptide (*Pthlh or PthrP*)^22^, was not significantly altered in *Smurf2*^*−/−*^ osteoblasts. Similar to the data from neonatal calvarial osteoblast cells, osteoblasts from the long bones of 5-week-old *Smurf2*^*−/−*^ mice showed increased *Rankl* expression, but the expression of *Opg* and other osteoclast differentiation regulators was unchanged (Supplementary Fig. 2E). Concurrently, *Smurf2*^*−/−*^ mice displayed higher serum levels of RANKL (Fig. 4o). The higher level of RANKL in *Smurf2*^*−/−*^ mice was also confirmed by immunoblotting using cultured primary calvarial osteoblasts (Fig. 4p). Taken together, these data indicate that *Smurf2*-deficient osteoblast cells augment osteoclastogenesis by increasing the expression of *RANKL*.

### SMAD3 ubiquitination inhibits RANKL expression

**Figure 5:**
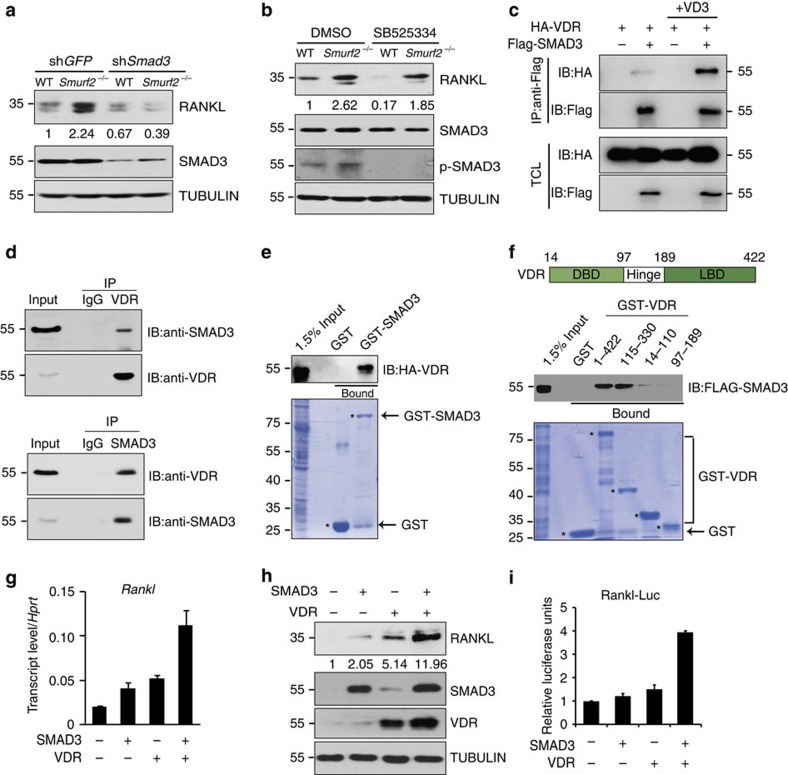
SMAD3 and VDR function together to induce RANKL expression. (**a**) Expression of RANKL, SMAD3 and TUBULIN in WT and *Smurf2*^*−/−*^ neonatal calvarial osteoblasts infected with shRNA lentivirus targeting *Smad3* or *Egfp* shRNA. (**b**) Expression of RANKL, SMAD3, p-SMAD3 and TUBULIN in WT and *Smurf2*^*−/−*^ neonatal calvarial osteoblasts with or without 10 μM SB525334. (**c**) Co-immunoprecipitation of HA-VDR and Flag SMAD3 in 293T cells with dimethylsulphoxide (DMSO) or 10 nM 1,25D treatment for 24 h. (**d**) Co-immunoprecipitation of endogenous VDR and SMAD3 in calvarial osteoblasts. (**e**,**f**) 293T cells were transfected with the HA-VDR or Flag SMAD3 construct, and 48 h post transfection, total cell lysates (TCLs) were recovered to perform GST pull-down analysis with the indicated GST-fusion proteins. (**g**,**h**) mRNA and protein level of RANKL in C3H10 T1/2 cells infected with SMAD3- and VDR-expressing lentivirus. (**i**) C3H10 T1/2 cells were transiently co-transfected with a RANKL 2-kb promoter luciferase reporter and pRL-Renilla plasmids together with empty vector or Smad3 or VDR. The cells were collected for dual-luciferase reporter assay after transfection for 48 h.

We next sought to investigate the mechanism in which SMURF2 regulates RANKL expression. The WW domains of SMURF2 interact with the PPxY motif of its substrate and dozens of SMURF2-interacting proteins have been reported. We therefore examined whether proteins known to interact with SMURF2 could affect RANKL expression and found that NEK6, SERTAD2 and SMAD3 could increase RANKL expression when C3H10 T1/2 cells were infected with lentivirus expressing these proteins (Supplementary Fig. 3A–C). Next we determined whether these three proteins are ubquitination substrates of SMURF2. As shown in Supplementary Fig. 3D–F, SMAD3, but not NEK6 and SERTAD2, could be ubiquitinated by SMURF2, consistent with the previous reports that SMAD3 is a major substrate of SMURF2-mediated ubiquitination^23^,^24^,^25^,^26^. These findings led us to pursue the possibility that SMURF2 regulates RANKL expression by controlling the signalling pathways related to SMAD3, a member of the family of receptor-regulated Smad proteins. To examine whether the higher RANKL levels in *Smurf2*^*−/−*^ osteoblasts are related to SMAD3 ubiquitination, we asked whether knockdown of *Smad3* interfered with the increased RANKL levels in *Smurf2*^*−/−*^ osteoblasts. As shown in Fig. 5a, knockdown of *Smad3* decreased RANKL levels in both WT and *Smurf2*^*−/−*^ osteoblasts. Moreover, Smad3 deficiency eliminated increased RANKL production by *Smurf2*^*−/−*^ osteoblasts, indicating that the regulation of RANKL expression in *Smurf2*^*−/−*^ mice is SMAD3 dependent (Fig. 5a). It was reported previously that SMURF2 negatively regulates TGF/Smad signalling either by inducing the degradation of Smads^27^,^28^,^29^,^30^ or by interfering with the formation of homotrimeric or heterotrimeric SMAD3 complexes via the ubiquitination of SMAD3 (ref. 26). We next treated primary calvarial osteoblasts with the TGF-β signalling inhibitor SB525334 and found that the level of RANKL could be reduced by SB525334 in both WT and *Smurf2*^*−/−*^ osteoblasts. However, *Smurf2*^*−/−*^ osteoblasts still displayed higher RANKL expression than WT cells after SB525334 treatment (Fig. 5b), indicating that elevated TGF-β signalling is not responsible for the higher *RANKL* expression in *Smurf2*^*−/−*^ osteoblasts. This is consistent with the lack of Smad-binding elements in the proximal *RANKL* promoter^24^,^31^. The SMAD3 dependence of increased *RANKL* expression in *Smurf2*^*−/−*^ mice suggested that another Smad3-binding transcription factor might control *RANKL* expression. RUNX2, vitamin D receptor (VDR) and CCAAT/enhancer-binding protein directly bind to the RANKL promoter and regulate *RANKL* expression. Among these, VDR is a member of the nuclear hormone receptor superfamily of transcriptional regulators and mediates the diverse biological effects of calcitriol (1,25-(OH)_2_D_3_) and its analogues^32^,^33^. It has also been widely studied for its therapeutic potential in treating various forms of skeletal pathology^34^,^35^,^36^. Previous studies reported five mouse and human vitamin D response elements (VDREs) in the enhancer regions upstream of the *RANKL* transcriptional start site^11^,^37^,^38^,^39^. We confirmed the interaction between SMAD3 and VDR, as previously reported^40^, by immunoprecipitation of FLAG epitope-tagged SMAD3 followed by immunoblotting with an anti-HA antibody to detect HA-tagged VDR (Fig. 5c). The interaction between endogenous SMAD3 and VDR was also confirmed in calvarial osteoblast cells *in vivo* (Fig. 5d). The interaction between SMAD3 and VDR is direct as it can be recapitulated in a cell-free system using recombinant proteins (Fig. 5e,f). We next examined whether SMAD3 and VDR can function together to induce *RANKL* expression. As shown in Fig. 5g,h, simultaneous infection of C3H10 T1/2 with SMAD3- and VDR-expressing lentivirus led to an elevation in *RANKL* expression. We also examined the activity of Rankl promoter using luciferase reporter assay. Consistently, VDR and SMAD3 could additively increase the activity of Rankl promoter (Fig. 5i). This Rankl promoter contains VDR-binding element but does not contain SMAD3-binding element. These data suggest that SMAD3 could additively increase RANKL expression through directly interacting with VDR, which binds to RANKL promoter and increases RANKL expression.

**Figure 6:**
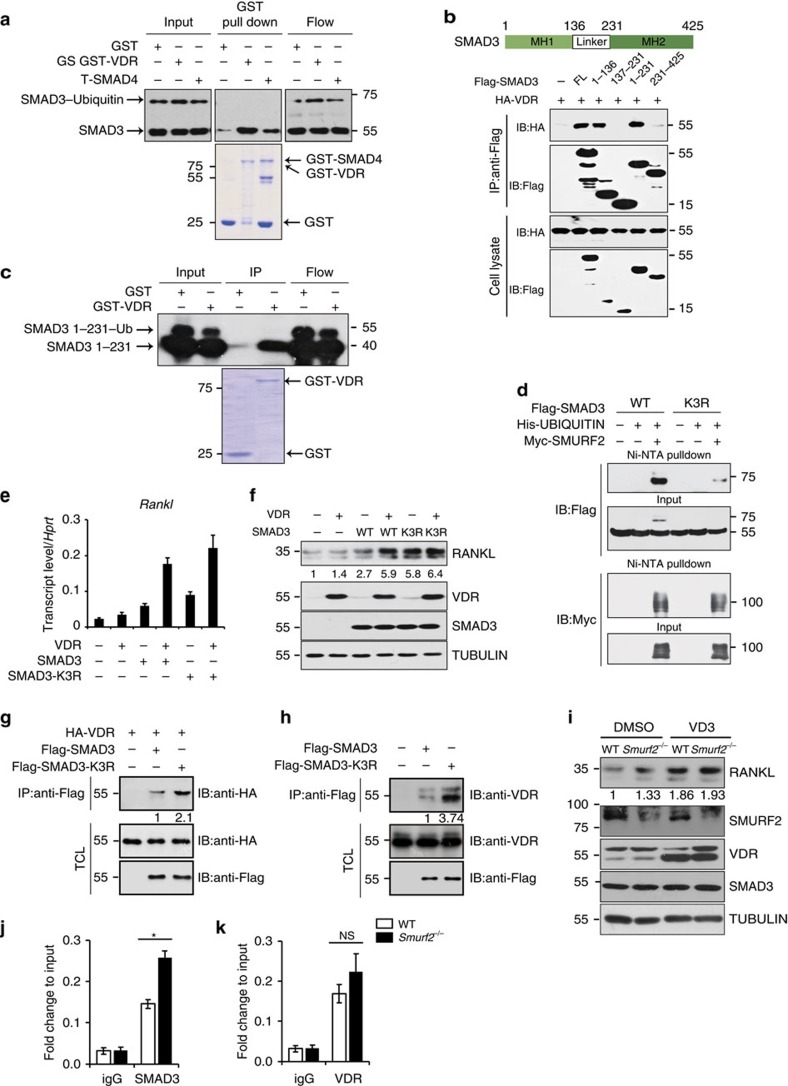
SMURF2-mediated SMAD3 monoubiquitination impedes formation of the SMAD3–VDR complex. (**a**) Ubiquitinated and non-ubiquitinated HA-SMAD3 prepared from transfected 293T cells were performed GST pull-down experiments with the indicated GST-fusion proteins. (**b**) Immunoblot analysis of total cell lysates (TCLs) derived from 293T cells transfected with HA-VDR and the indicated Flag SMAD3 constructs. (**c**) Ubiquitinated and non-ubiquitinated HA-SMAD3 1–231 truncations prepared from transfected 293T cells were performed GST pull-down experiments with the indicated GST-fusion proteins. (**d**) Ubiquitination analysis of SMAD3 and N-terminal ubiquitin acceptor site lysine mutants in 293T cells. (**e**,**f**) mRNA and protein level of RANKL in C3H10 T1/2 cell line infected with lentivirus-expressing SMAD3, SMAD3-K3R and VDR. (**g**) Co-immunoprecipitation experiments were conducted in 293T cells transfected with Flag SMAD3/SMAD3-K3R and HA-VDR. (**h**) Co-immunoprecipitation of Flag SMAD3/SMAD3-K3R with endogenous VDR in calvarial osteoblast cells infected with lentivirus-expressing SMAD3/SMAD3-K3R mutant protein. (**i**) Immunoblot for RANKL, SMURF2, VDR and TUBULIN in response to 10 nM 1,25D treatment for 24 h in WT and *Smurf2*^*−/−*^ calvarial osteoblasts. (**j**,**k**) WT and *Smurf2*^*−/−*^ calvarial osteoblasts were subjected to immunoprecipitation with IgG, SMAD3 or VDR antibody. Quantitative PCR analysis of DNA precipitates to amplify VDRE site in the mouse Rankl promoter. Three sets of independent experiments were performed to generate each data and the error bars represent mean±s.d. (*n*=3 for each genotype, *P<0.05, NS, not significant. Student's *t*-test).

As SMAD3 can interact with VDR and additively augment the expression of *RANKL* in osteoblasts, we examined whether SMURF2-mediated SMAD3 monoubiquitination could block the formation of the SMAD3–VDR complex. To test whether ubiquitination imposes a steric hindrance to the interaction of SMAD3 and VDR, we co-transfected plasmids expressing SMURF2, ubiquitin and SMAD3 into HEK-293T cells to give rise to both an unmodified 55 kDa and an ubiquitin-modified 72 kDa SMAD3 protein product, then extracted SMAD3 proteins to conduct *in vitro* interaction analysis using purified VDR. This demonstrated that only the unmodified 55 kDa but not the modified 72 kDa SMAD3 was able to interact with recombinant VDR (Fig. 6a). Recombinant SMAD4 was used as a control in this assay. These data indicate that ubiquitination of SMAD3 disrupts the interaction between SMAD3 and VDR. Ubiquitin conjugation occurs at Lys ɛ-amino groups of targeted proteins. Since our study and others^40^,^41^ suggested that the SMAD3 MH1 domain interacts with VDR (Fig. 6b), we next determined whether the MH1 domain can be modified by SMURF2-induced ubiquitination. The linker domain of SMAD3 contains a PY motif, which directly interacts with SMURF2 (Supplementary Fig. 4A,B). While the linker domain fragment of SMAD3 could not be ubiquitinated, SMAD3 1–231, which contains both the MH1 and linker domains, can be ubiquitinated, and this ubiquitination can be augmented by SMURF2 (Supplementary Fig. 4C,D), indicating that the MH1 domain contains ubiquitination sites. Moreover, the interaction between VDR and the SMAD3 MH1 domain was also blocked by SMAD3 ubiquitination (Fig. 6c), indicating that ubiquitination interfered with the binding between SMAD3 and VDR. Previous studies have demonstrated that K33, K53 and K81 within the SMAD3 MH1 domain are important sites for monoubiquitination^42^. We constructed a SMAD3 mutant (SMAD3-K3R), where K33, K53 and K81 were substituted with arginine and were therefore unsuitable for ubiquitin attachment. As shown in Fig. 6d, SMAD3-K3R mutation led to a marked reduction in the level of SMURF2-mediated ubiquitination, and showed a stronger interaction with VDR when it influence on *RANKL* expression compared to WT SMAD3 (Fig. 6e,f). Consistently, SMAD3-K3R showed increased interaction with exogenous VDR protein in 293T cells compared to the WT SMAD3 (Fig. 6g). Similarly, more endogenous VDR proteins were co-immunoprecipitated by exogenous SMAD3-K3R compared to the WT SMAD3 in calvarial osteoblasts (Fig. 6h). We next treated both WT and *Smurf2*^*−/−*^ primary calvarial osteoblasts with 1,25-(OH)_2_D_3_. As shown in Fig. 6i, 1,25D treatment could increase levels of VDR as previously reported. Furthermore, we found that RANKL levels were augmented by 1,25D in WT osteoblasts. However, 1,25D treatment failed to further augment RANKL levels in *Smurf2*^*−/−*^ osteoblasts (Fig. 6i). The resistance to RANKL induction incurred by 1,25D treatment in *Smurf2*^*−/−*^ osteoblasts indicates enhanced SMAD3-related VDR signalling. To test whether ubiquitination influences recruitment of VDR and SMAD3 to the VDREs, we performed chromatin immunoprecipitation (ChIP) assays and found that SMURF2 deficiency increased recruitment of SMAD3 to the portion of the *RANKL* promoter covering the VDREs (Fig. 6j); however, there was no significant difference in VDR recruitment between WT and *Smurf2*^*−/−*^ osteoblasts (Fig. 6k). Hence, we conclude that SMURF2-mediated SMAD3 monoubiquitination interferes with the formation of a SMAD3–VDR complex, hence preventing *RANKL* induction by this complex.

### *Smurf2* mesenchymal conditional KO mice have osteopenia

**Figure 7:**
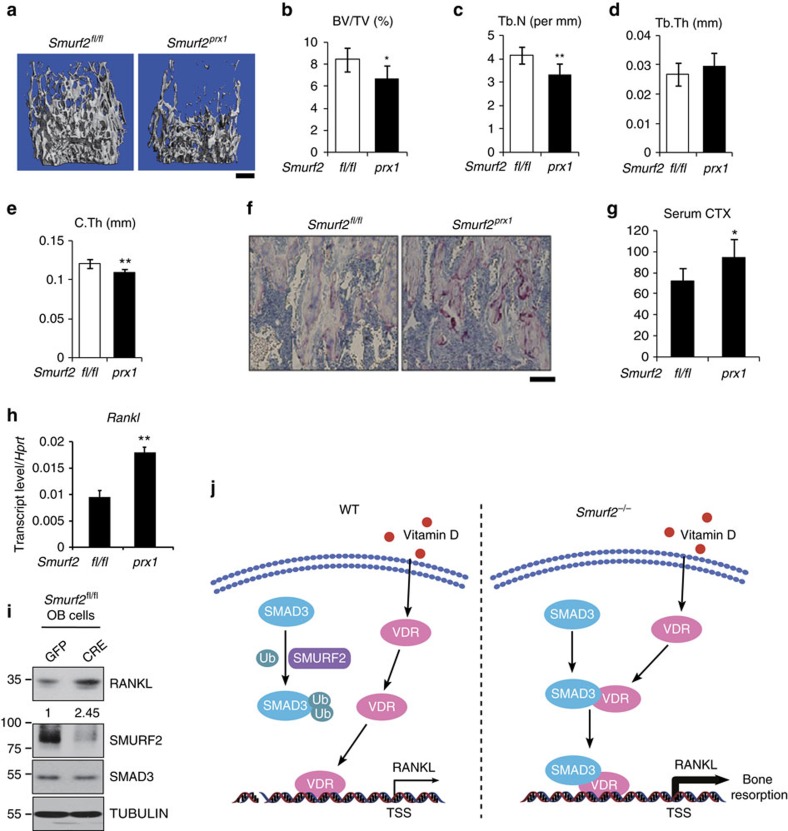
Deletion of *Smurf2* in mesenchymal cells leads to increased bone resorption. (**a**–**e**) μ-QCT analysis of proximal femur from 5-week-old *Smurf2*^fl/fl^ and *Smurf2*^prx1^ male mice for bone volume per tissue volume (BV/TV), trabecular number (Tb.N), trabecular thickness (Tb.Th) and cortical thickness (C.Th). Scale bar, 500 μm. (**f**) Histological sections of tibias from 5-week-old *Smurf2*^fl/fl^ and *Smurf2*^prx1^ male mice stained for tartrate-resistant acid phosphatase (TRAP). Scale bar, 100 μm. (**g**) Serum CTX levels in 5-week-old *Smurf2*^fl/fl^ and *Smurf2*^prx1^ male mice. Value represent means±s.d. (*n*=6 for each genotype, **P<0.01, *P<0.05, NS, not significant. Student's *t*-test). (**h**) Quantitative PCR analysis of *Rankl* expression in the bone of *Smurf2*^prx1^ compared to the *Smurf2*^fl/fl^ male mice. Values represent means±s.d. (*n*=3 for each genotype, **P<0.01, *P<0.05, Student's *t*-test). (**i**) Expression of RANKL, SMURF2, SMAD3 and TUBULIN in *Smurf2*^fl/fl^ calvarial osteoblast cells infected with the lentivirus-expressing GFP and CRE recombinase. (**j**) Model depicting the mechanism through which SMURF2-mediated monoubiquitination of SMAD3 leads to decreased RANKL expression.

To further explore the mechanism whereby SMURF2 controls the coupling between bone formation and resorption *in vivo*, a mice line harbouring a *Smurf2* allele in which exon 14 is flanked by loxP sites were generated (Supplementary Fig. 5A,B), hereafter called *Smurf2*^F/F^ mice. We intercrossed *Smurf2*^F/F^ mice with *Prx1-Cre* expressing mice, which express CRE recombinase in the mesenchyme of the limbs and skull but not in the axial skeleton^43^. We collected RNA from femurs and found reduced *Smurf2* messenger RNA (mRNA) levels in *Prx1-Cre Smurf2*^F/F^ mice as expected (Supplementary Fig. 5C). μ-QCT analysis demonstrated that *Prx1-Cre Smurf2*^F/F^ mice showed lower femoral bone mass than *Smurf2*^F/F^ controls (Fig. 7a–e). Thus, the bone mass phenotype of *Smurf2* mesenchymal conditional knockout (KO) mice was similar to that of germline *Smurf2*^*−/−*^ mice. On the basis of our proposed model that *Smurf2* deficiency in the mesenchymal/osteoblast lineage augments *RANKL* expression, leading to increased bone resorption, we expected that *Prx1-Cre Smurf2*^F/F^ mice would show increased osteoclast numbers and activity. Indeed, histology demonstrated that *Prx1-Cre Smurf2*^F/F^ mice displayed increased numbers of osteoclasts (Fig. 7f) and increased CTX levels (Fig. 7g), indicating elevated systemic bone resorption activity. We also found increased *RANKL* expression in the bones of *Prx1-Cre Smurf2*^F/F^ mice compared with *Smurf2*^F/F^ mice by quantitative PCR (Fig. 7h). RANKL levels are known to change during the course of osteoblast differentiation. To assess the contribution of osteoblast differentiation states to differences in RANKL levels, we cultured calvarial osteoblasts from *Smurf2*^F/F^ mice and then infected the cells with lentivirus-expressing GFP and CRE recombinase. As shown in Fig. 7i, CRE lentivirus led to reduced SMURF2 expression accompanied by higher RANKL levels. When the cells were cultured in non-differentiation media for 48 h after the virus infection, there was no difference in osteoblast differentiation determined by osteoblast gene expression, but Cre virus induced knockout of Smurf2 mRNA accompanied with increased Rankl expression (Supplementary Fig. 5D). These data indicated that the effects of Smurf2 on Rankl expression are not the results of the generalized increase in the differentiation stage. When the cells were cultured in osteoblast differentiation media for 4 days, there were increased osteoblast differentiation in Cre-infected cells and the increase of Rankl expression are kept (Supplementary Fig. 5E), it is hard to distinguish the effects of Smurf2 itself from the effects of generalized increase in differentiation due to the limitations of the experiments. Taken together, these findings support our model that SMURF2 in osteoblast lineage cells directly controls the expression of *RANKL* through interfering with the Smad3–VDR complex to regulate osteoclastic bone resorption (Fig. 7j).

## Discussion

SMURF1 and SMURF2 are a C2-WW-HECT class of ubiquitin E3 ligases that play important roles in regulating cell signalling, cell polarity and motility^16^,^44^,^45^. Multiple lines of evidence including their similar expression patterns, high degree of amino-acid sequence homology, and the ability to promote ubiquitination of the same targets^14^,^15^,^26^,^27^,^29^,^46^ suggests that SMURF1 and SMURF2 display partial functional redundancy. The embryonic lethality of *Smurf1*/*Smurf2* double-deficient mice provides definitive genetic evidence of their functional redundancy^16^. Hence, our finding that *Smurf2*^*−/−*^ animals display decreased bone mass, opposite to the skeletal phenotype of *Smurf1*-deficient mice, is unexpected and suggests novel SMURF2 functions. We performed osteoblast differentiation assays with primary human mesenchymal stem cells and examined the effects of SMURF2 and SMURF1 on osteoblast differentiation and RANKL expression. As expected, knockdown of either *Smurf1 or Smurf2* in human mesenchymal stem cells increased osteoblast differentiation as determined by ALP and alizarin red staining. However, only *Smurf2* shRNAs, but not *Smurf1* shRNAs, increased *Rankl* expression. Thus, while SMURF1 and SMURF2 have overlapping functions to inhibit osteoblast differentiation, their effects on RANKL expression diverge (Supplementary Fig. 6).

Our current study suggests that SMURF2 regulates osteoblast-dependent osteoclastogenesis by controlling *RANKL* expression through SMAD3 ubiquitination-related VDR signalling, a distinct function not likely to be shared by SMURF1, which negatively regulates osteoblast activity through controlling MEKK2 ubiquitination. Huang *et al*.^47^ demonstrated that there are no differences in cortical, subchondral and trabecular bone between WT and homozygous *Smurf2* gene-trap mice at 4 or 16–24 months of age. We believe that the divergent results between their study and the current report likely reflect the clear presence of residual levels of SMURF2 in *Smurf2* gene-trap mice^48^.

Ubiquitination is a major mechanism for controlling protein functional activity and physiologic responses^49^. Previous studies identified a long list of Smurf ubiquitin substrates, including SMADs1, 2/3, 5, 6/7, type 1 TGF-β and BMP receptors, MEKK2 (ref. 15), Par6 (ref. 50), Gsk3 (ref. 51), Connexin 43 (ref. 52), RhoA (ref. 45), KLF5 (ref. 53) and others. Ectopic expression of SMURF1 and/or SMURF2 can degrade and destabilize these substrates. However, conflicting reports have emerged as to whether SMURF1 and/or SMURF2 can induce the ubiquitination and degradation of these proteins under physiological conditions^26^. Two independent studies failed to detect changes in SMAD2, SMAD3 or type I receptor protein levels in either *Smurf1* or *Smurf2*-deficient primary fibroblast cells^15^,^26^. Instead of polyubiquitination and degradation, SMURF2 induced the multiple-site monoubiquitination of SMAD3 *in vivo* and inhibited the nuclear accumulation of the SMAD3 complex^26^. Here we confirmed that SMURF2 mediates monoubiquitination of SMAD3. As with the SMAD3/SMAD4 complex, monoubiquitination of SMAD3 impedes the formation of a SMAD3–VDR complex, which promotes the expression of *RANKL* in osteoblasts. VDR is a member of the nuclear receptor family of transcription factors and induces *RANKL* upregulation by vitamin D, 1,25-dihydroxyvitamin D3. There are five sites of VDR dimer binding 16, 22, 60, 69 and 76 kb upstream of the *RANKL* transcriptional start site^11^. Consistent with the existence of VDR-binding sites, monoubiquitination of SMAD3 that disrupts the VDR complex inhibits *RANKL* signalling. Hence, our study identifies SMURF2 as a regulator of osteoclastogenesis and bone homeostasis that act upstream of RANKL signalling.

To address how SMURF2 expression is regulated in osteoblasts, we treated preosteoblast cells with TGF-β, BMP2 and oestrogen. As shown in Supplementary Fig. 7, TGF-β, but not BMP2 or oestrogen, regulates *Smurf2* expression. This regulation of *Smurf2* expression by TGF-β can increase *Smurf2* expression in a dose- and time-dependent manner. TGF-β is widely known to play critical roles in cellular proliferation and differentiation through receptor-regulated SMAD proteins such as SMAD2 and SMAD3, which are phosphorylated upon type I and type II cell surface receptor activation and in turn bind to SMAD4 and translocate into the nucleus, thus propagating the signal^54^. The TGF-β-activated transcriptional programme is involved in various pathological and physiological processes^55^. In particular, TGF-β is abundant in bone matrix and its pivotal role in bone development and remodelling has been addressed^56^. A rich reservoir of TGF-β in the bone milieu can enhance the expression of multiple factors such as PTH-like hormone^22^ and JAG1 (Jagged 1)^57^ that tip the balance of bone remodelling in favour of osteolysis^58^. In the setting of osteolytic bone metastases, this process may generate a vicious cycle promoting feed-forward destruction of bone. Although several prominent pathways such as PI3K, MAPK^59^, Rho/RAC1 (ref. 60) and Notch have been shown to regulate bone homeostasis by TGF-β, the exact function of TGF-β during OB differentiation and many other developmental aspects remains unresolved. The regulation of Smurf2 expression by TGF-β is consistent with the previous work, showing that TGF-β stimulates Smurf2 expression by Smad-independent pathways such as the PI3K/Akt pathway via the TGF-β receptor^61^. This regulation of SMURF2 expression by TGF-β suggests that the role of SMURF2 in preventing excessive OC formation may be also regulated by TGF-β.

Liphophilic hormone vitamin D exerts important biological functions on the maintenance of calcium and bone homeostasis through activating VDR, which is a ligand-dependent transcription factor belonging to the nuclear receptor superfamily^62^,^63^. VDR mediates 1,25(OH)2D function via heterodimerizing with the retinoid X receptor and consequently interacting with response elements in the promoter regions of target genes, including Ca metabolism mediators such as TRPV6 (ref. 64). The role of VDR itself has been examined by using an osteoblast-specific VDR-knockout (KO; VDR^ΔOb/ΔOb^) mouse, generated with 2.3 kb-1(I)-collagen promoter-Cre transgenic mice and VDR fl/fl mice^65^. VDR^ΔOb/ΔOb^ mice displayed higher bone density due to the decreased bone resorption, although mineral metabolism parameters and bone formation parameters were not affected. More interestingly, VDR^ΔOb/ΔOb^ mice demonstrated lower RANKL expression. The phenotype and the mechanism of VDR^ΔOb/ΔOb^ mice are consistent with our model. In the *Smurf2*^*−/−*^ mice, the amount of SMAD3 that binds to VDR is increased because of the loss of monoubiquitination of SMAD3. Overall, our model is that Smurf2 KO mice displayed increased VDR-induced RANKL expression. However, the dependence of vitamin D in the function of VDR in our model needs further investigation to determine.

Crosstalk between TGF-β and vitamin D signalling pathways has been suggested by their cooperative actions on osteoblasts^66^. Explaining these observations, TGF-β treatment can enhance the transactivation activity of VDR through the SMAD3/VDR interaction^40^. Synergy between SMAD3 and VDR was also observed to regulate the human osteocalcin promoter in transient transfection experiments. In contrast to these synergistically cooperative actions, some reports reveal antagonistic actions between vitamin D and TGF-β^67^. Recent studies claim that VDR ligands inhibit TGF-β1/SMAD-induced activation of hepatic stellate cells and improve liver fibrosis in mice^67^. Mechanistically, VDR ligands could facilitate VDR binding to VDR/SMAD coregulated genes and reduce SMAD3 occupancy at these sites, leading to the inhibition of fibrosis^67^. Overall, these synergistic or antagonistic actions between vitamin D and TGF-β could occur in a tissue-specific manner. The tissue-specific gene expression pattern and amounts of Smad(s), VDR and their receptors may be responsible for tissue-specific differences in cooperative actions between SMADs and VDR.

Bone mass is under sophisticated control of bone formation by endocrine and paracrine signals, and bone resorption through osteoblast-dependent or haematopoietic lineage-autonomous mechanisms. Our finding shows that deletion of *Smurf2* in osteoblast affects both aspects of bone homeostasis. Thus, SMURF2 in osteoblasts may serve as a rheostat for bone homeostasis by coordinating the activities of osteoblasts and osteoclasts to regulate bone mass. The role of *Smurf2* in osteoblasts is similar to that observed in *Smurf1* loss-of-function studies in terms of increased osteoblast differentiation^15^. However, the *in vivo* phenotype of *Smurf1*^*−/−*^ and *Smurf2*^*−/−*^ mice is divergent revealing that SMURF2 and SMURF1 have non-overlapping and in some respects diametrically opposite functions.

## Methods

### Mice

*Smurf2*^*−/−*^ mice were from Dr Jeffery Wrana's lab and were previously described^16^. Conditional *Smurf2*^*F/F*^ mice, in which exon 14 of *Smurf2* allele is flanked by loxP sites, were generated at Merck and on a C57/BL6 background. Prx-1-Cre mice were purchased from the Jackson Laboratory. The mutant mice were validated by PCR-based DNA sequencing analysis and by western blot for SMURF2 protein expression. All animals were housed under specific pathogen-free conditions. All experiments using mice were approved by the Institutional Animal Care and Research Advisory Committee of the Shanghai Institute of Biochemistry and Cell Biology.

### Cell culture

293T cells (American Type Culture Collection) were maintained in DMEM containing 10% fetal bovine serum (FBS). C3H10 T1/2 cells (American Type Culture Collection) were maintained in α-MEM culture medium (containing 10% FBS, 2 mM L-glutamine(Corning), 1 mM sodium pyruvate (Corning), 10 mM Hepes buffer (Corning), 1% MEM nonessential amino acids (Corning) and 1% penicillin/streptomycin (Gibco)). Primary murine calvarial osteoblasts were isolated from 5-day-old neonates by collagenase/dispase II digestion. Calvarial bones were incubated in a-MEM containing 1 mg ml^−1^ collagenase (Sigma), 2 mg ml^−1^ dispase II (Roche) and 2% penicillin/streptomycin. The bones were placed in a shaker at 37 °C. Cells were released by 10 min of sequential digestions. The first digestions were discarded, and subsequent three digestions were collected. Cells released from digestion were plated in α-MEM culture medium and differentiated with 50 μg ml^−1^ L-ascorbic acid (Sigma) and 10 mM β-glycerophosphate (Sigma). For *in vitro* gene inactivation, floxed osteoblasts were infected with either GFP- or CRE-expressing lentivirus. For Von Kossa staining, cells were fixed at day 21 of culture with 10% neutral formalin buffer and stained with 5% silver nitrate for 30 min. For ALP assay, cells were fixed with 10% neutral buffered formalin and subjected to ALP staining on day 7.

### *In vitro* osteoclast differentiation

Mouse BM cells isolated from the femur and tibia were seeded (1 × 10^6^ cells per well in a 24-well plate) and cultured in α-MEM with 10% FBS containing 10 ng ml^−1^ M-CSF (PeproTech) for 3 days. Subsequently, nonadherent cells were discarded and adherent BMMs were further cultured in the presence of 20 ng ml^−1^ M-CSF and 150 ng ml^−1^ soluble RANKL. Cells were then fixed and stained for the presence of TRAP using a kit according to the manufacturer's instructions (Sigma-Aldrich).

### Co-culture experiments

Mouse calvarial osteoblast cells from WT and *Smurf2*^*−/−*^ mice were seeded equally into a 24-well plate (8 × 10^4^ cells per well) with α-MEM+10% FBS, osteoclast precursors (monocytes) were isolated as mentioned above and cultured for 24 h. The nonadherent BM cells (1 × 10^6^ cells per well in a 24-well plate) were collected and cultured with calvarial osteoblasts in the presence of 1,25-dihydroxyvitamin D3 (10 nM; Sigma-Aldrich) and PGE2 (1 uM; Sigma-Aldrich) and the culture medium was half-changed every 2 days. Osteoclasts were identified by TRAP staining^68^,^69^.

### Antibodies and reagents

Anti-RANKL antibody (ab45039; 1:1,000) was obtained from Abcam. Anti-SMAD3 (C67H9) antibody (9523, s; 1:1,000) and anti P-SMAD3 (S423/425) antibody (9520, s; 1:1,000) were purchased from Cell Signaling Technology. Monoclonal anti-HA antibody (SC-7392; 1:2,000), anti-VDR rabbit polyclonal antibody (SC-1008; 1:1,000), anti-VDR mouse monoclonal antibody (SC-13133; 1:1,000) and anti-TUBULIN antibody (SC-23948; 1:10,000) was from Santa Cruz Biotechnology. Anti-SMURF2 antibody (ABS59; 1:500) was obtained from Millipore. Monoclonal anti-FLAG antibody (F3165; 1:5,000) was from Sigma-Aldrich. Monoclonal anti-MYC antibody (AE010; 1:2,000) was from Abclonal Technology. Monoclonal anti-GFP antibody (66002-1; 1:10,000) was purchased from Proteintech. Rabbit IgG (SC-2027) was purchased from Santa Cruz Biotechnology and Mouse IgG (I5381) was from Sigma-Aldrich. Recombinant mouse TGF-β1 (catalogue no.5231) was purchased from Cell Signaling Technology; recombinant human BMP-2(120-02) was from Proteintech; β-oestradiol (oestrogen; 02194565.2) was from MP. SB525334 was purchased from Selleckchem.

### Skeletal preparation and staining

Mice were eviscerated and the skin was removed, and the resulting samples were transferred into acetone for 48 h after overnight fixation in 95% ethanol. Skeletons were then stained in Alcian blue and Alizarin red S solution and sequentially cleared in 1% KOH/20% glycerol.

### Histology and immunostaining

Limb tissues were skinned and fixed in 4% paraformaldehyde in PBS. Then, specimens were decalcified until soft and pliable. Dehydration of tissues was done by passage through graded ethanol, cleared twice in xylene and embedded in paraffin. Tissue sections (6 μm) were used for haematoxylin–eosin staining or TRAP staining according to the standard protocol. Digoxigenine (DIG)-labelled RNA probes were generated according to the manufacturer's protocol. DIG-labelled antisense probes were generated to detect Col1a1 mRNA expression. Probes were then hybridized with paraffin sections and visualized using an anti-DIG horseradish peroxidase conjugate system.

### μ-CT analysis

The mouse femurs were collected, soft tissues were removed and the remaining tissues were fixed in 70% ethanol and μ-CT analysis scanned a region 0.28 mm proximal to the distal growth plate using a Scanco Medical μ-CT 35 system, with a spatial resolution of 7 μm. The region selected for analysis was the region of 2.1 mm in length of distal metaphysis. The interesting region segmented by a fixed threshold was reconstructed into three-dimensional images. The related properties of trabecular bone, such as Tb.Th, Tb.N, BV/TV, C.Th, were then calculated according to distance transformation of the binarized images^70^.

### Whole-mount TRAP histochemistry

Calvariae were dissected from 4-week-old mice and fixed in 100% methanol for 5 min (ref. 8). Calvariae were washed in PBS and then TRAP staining was perfomed according to the instructions of the manufacturer (Sigma).

### Immunoblots and immunoprecipitation

Tissues were washed with chilled PBS and lysed in tissue lysis buffer (50 mM Tris, pH 7.4, 150 mM NaCl, 1% TritonX-100, 1 mM EDTA and 0.1% SDS) supplemented with protease inhibitors cocktail (Sigma) by automated tissue homogenate instrument (JXFSTPRP-48; Shanghai Jingxin Industrial Development Co., Ltd). Cells were washed with chilled PBS and lysed in EBC buffer (50 mM Tris, pH 7.5, 120 mM NaCl and 0.5% NP-40) supplemented with protease inhibitors cocktail (Sigma). Lysates were then separated by SDS–polyacrylamide gel electrophoresis and immunoblotted with the indicated antibodies. For co-immunoprecipitation, lysates were incubated with Anti-FLAG M2 Affinity Gel (Sigma F2426) or Anti-HA Agarose Fast Flow (AOGMA, AGM90054) at 4 °C overnight. Precipitates were washed three times with lysis buffer before being resolved by SDS–polyacrylamide gel electrophoresis and were immunoblotted with indicated antibodies.

Uncropped scans of all blots are provided as Supplementary Fig. 8.

### Ni-NTA agarose purification

Cells were collected at 48 h after plasmid transfection and were lysed in urea lysis buffer (8 M urea containing 0.5% Triton x-100, 10 mM imidazole and 34 mM β-mercaptoethanol). Ni-NTA agarose beads (Qiagen) were then incubated with cell extracts and rotated at room temperature for 4 h or overnight. Precipitates were washed five times with urea lysis buffer and then boiled in 1 × sample buffer.

### Real-time reverse transcriptase–PCR analysis

Total RNA was prepared using TRIzol (Sigma) and was reverse-transcribed into cDNA using the PrimeScript RT Reagent kit (TakaRa). The real-time reverse transcriptase–PCR reaction was performed using the Bio-Rad CFX96 system.

### Transient transfections and reporter gene assays

For transient transfections, C3H10 T1/2 cells were seeded overnight in a 12-well dish at a concentration of 8 × 10^4^ cells per well. Cells were then transfected with a Rankl luciferase reporter gene plasmid and varied combinations of VDR and SMAD3 expression constructs, as indicated, using Effectene transfection reagent (Qiagen). pRL-TK (Promega) was co-transfected as a normalization control for transfection efficiency. Forty-eight hours after transfection, luciferase assays were performed using the Dual-Luciferase Reporter Assay System (Promega). pRankl-Luc plasmid was made by inserting Mlu1/Bgl2-digested PCR products, which was amplified using former primer (5′-TTAATTacgcgtAAGAACTCTGTAGGAATGTAACATA-3′) and reverse primer (5′-TTAATTagatctTCCTGGGGCGCGCGGGCGA-3′), into Mlu1/Bgl2-digested luciferase plasmid pGL3-Basic.

### Serum measurements

Blood samples were collected from WT, *Smurf2* germline KO and *Smurf2* conditional KO mice. Serum RANKL was measured by using a RANKL Elisa kit (Sangon Biotech) according to the instructions of manufacturer.

### ChIP assays

ChIP was performed using the EZ ChIP kit (Millipore) according to the instructions of manufacturer. Recovered DNA samples were subjected to PCR amplification with forward primer (5′-AGGCAGAGGCGAGTGGAT-3′) and reverse primer (5′-TGTCGTTTTGGTTTATTTGTTTG-3′) to amplify the vitamin D responsive element binding site on the RANKL promoter region (−937/−922).

### Statistics

Statistical significance was determined by unpaired, two-tailed Student's *t*-test for comparison between two groups with a *P* value of <0.05 considered statistically significant.

### Data analysis

Experimental sample sizes required for adequate power were determined by the expected CVs we have previously observed with each assay, using the standard *β* value of 0.8 and *α* value of 0.05, and our sample sizes are similar to those generally employed in the field. All *n* values defined in the legends refer to biological replicates unless otherwise indicated. Studies were not blinded to investigators or formally randomized. No inclusion or exclusion criteria were used. Data distribution was assumed to be normal, but this was not formally tested^68^.

### Data availability

The authors declare that all data supporting the findings of this study are available within the article and its Supplementary Information files or are available from the authors upon reasonable request.

## Additional information

**How to cite this article:** Xu, Z. *et al*. SMURF2 regulates bone homeostasis by disrupting SMAD3 interaction with vitamin D receptor in osteoblasts. *Nat. Commun.* **8,** 14570 doi: 10.1038/ncomms14570 (2017).

**Publisher's note:** Springer Nature remains neutral with regard to jurisdictional claims in published maps and institutional affiliations.

## Supplementary Material

Supplementary Information Supplementary Figures and Supplementary Table
