# Supplementary material for: SMURF2 regulates bone homeostasis by disrupting SMAD3 interaction with vitamin D receptor in osteoblasts
Source: Nat Commun. 2017 Feb 20;8:14570. doi: 10.1038/ncomms14570 (PMC5321737; doi:10.1038/ncomms14570)
Supplement: Supplementary Information — Supplementary Figures and Supplementary Table [file ncomms14570-s1.pdf]

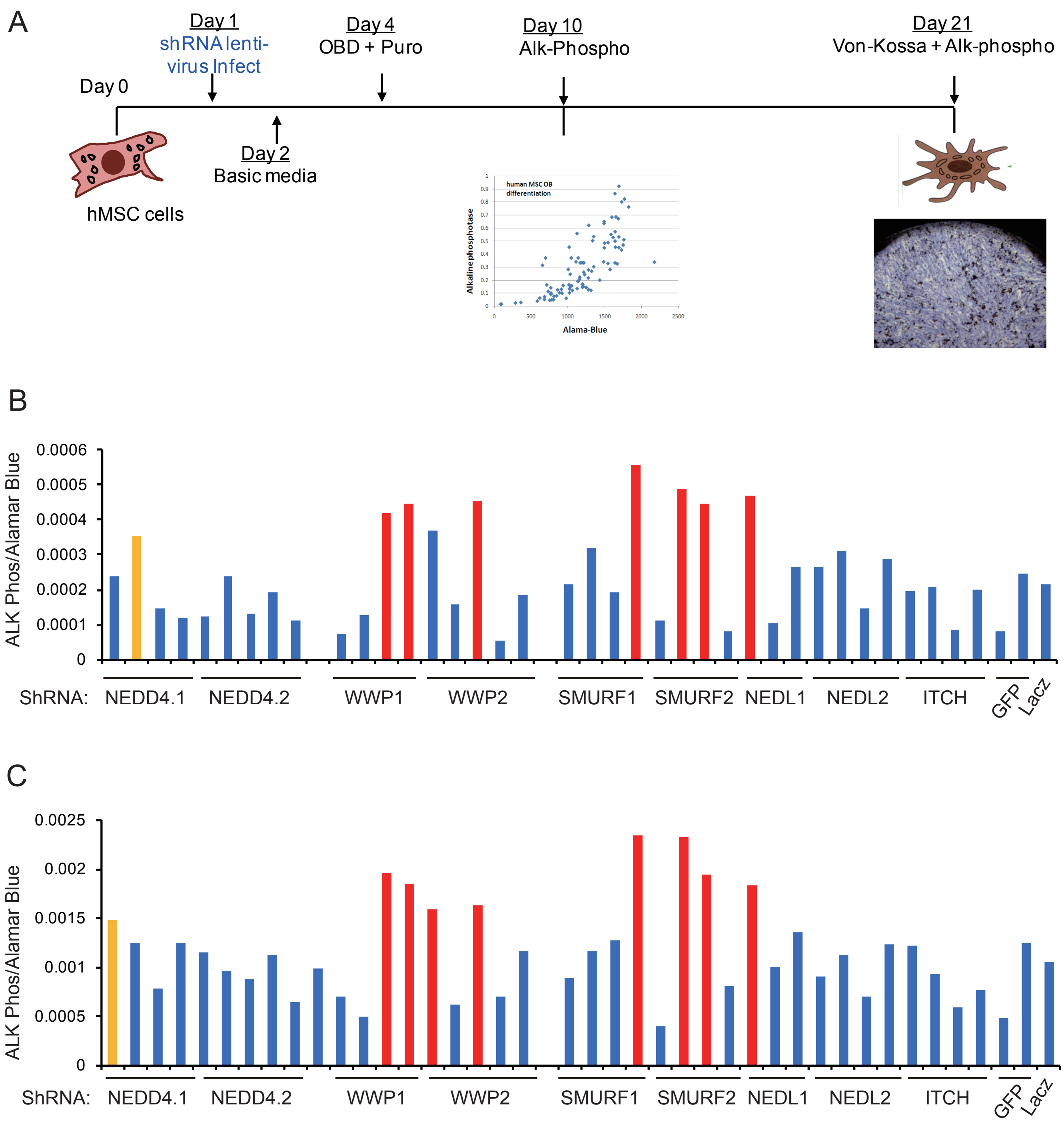

Supplementary Figure 1. RNAi-based loss-of-function screen in human mesenchymal stem cells. (A) Schematic of lentiviral based shRNA screen utilizing hMSCs. (B-C), Analysis of ALP in two independent experiments using hMSC cells derived from two different donors after infection with shRNAs targeting different members of NEDD4 E3 ligases, GFP and Lacz.

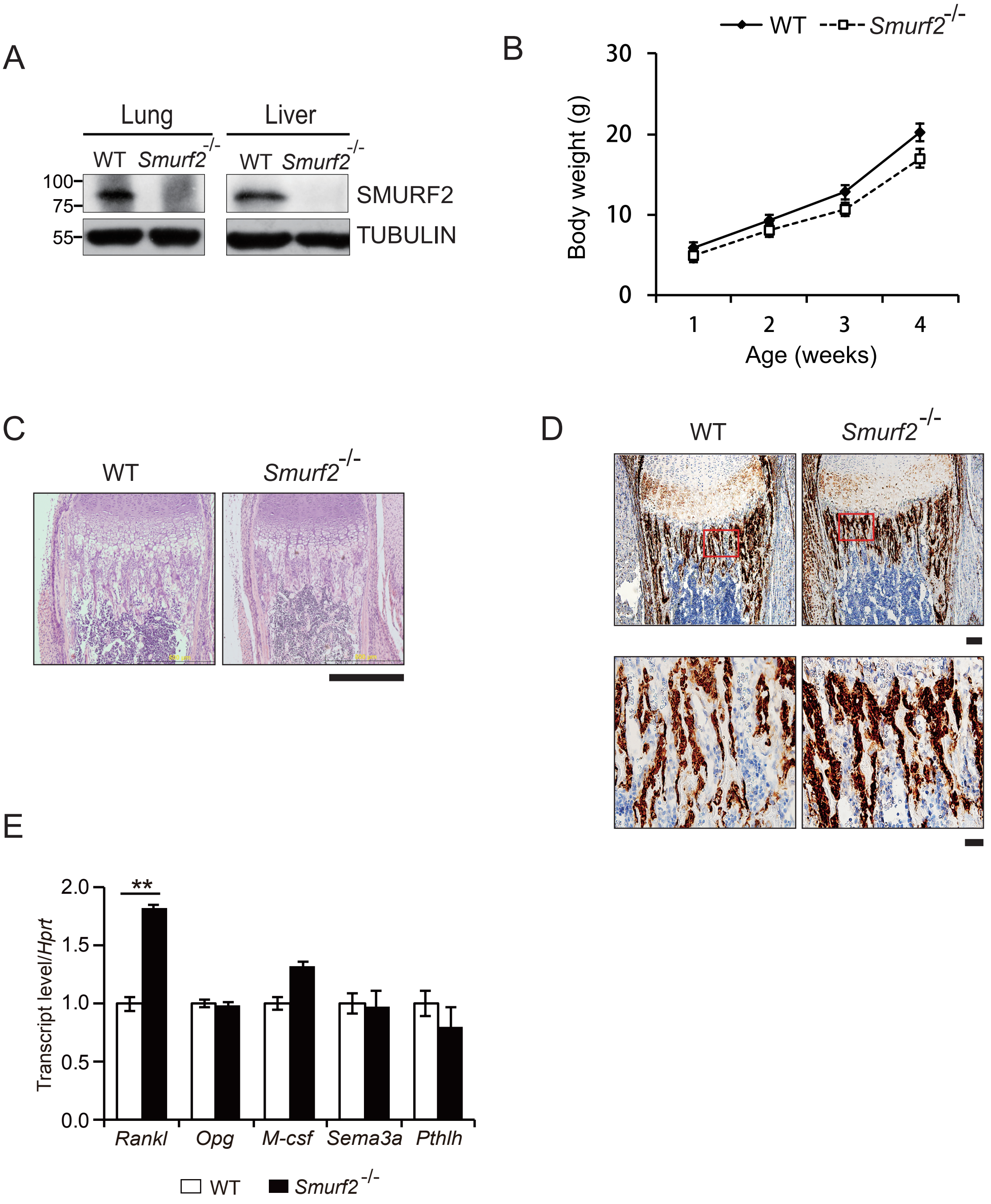

Supplementary Figure 2. The phenotype of global *Smurf2* knockout mice. (A) Immunoblot for SMURF2 and TUBULIN in lung and liver derived from the wild type and *Smurf2*$^{-/-}$ mice. (B) Body weight analysis of WT and *Smurf2*$^{-/-}$ male mice, n=10 for each genotype. (C) H&E staining of tibia of 1-week-old male WT and *Smurf2*$^{-/-}$ mice, Scale bar, 500μm. (D) In situ hybridization for collagen I(col I) in the tibia of 1-week-old male WT and *Smurf2*$^{-/-}$ mice, (upper,scale bar, 100μm;bottom,scale bar 25μm). (E) Gene expression of major osteoclast differentiation regulators in osteoblast cells from long bone marrow of 5-week-old male mice. All data are the mean±s.d. *P<0.05 , **P<0.01, ***P<0.001.Student's t-test was performed.

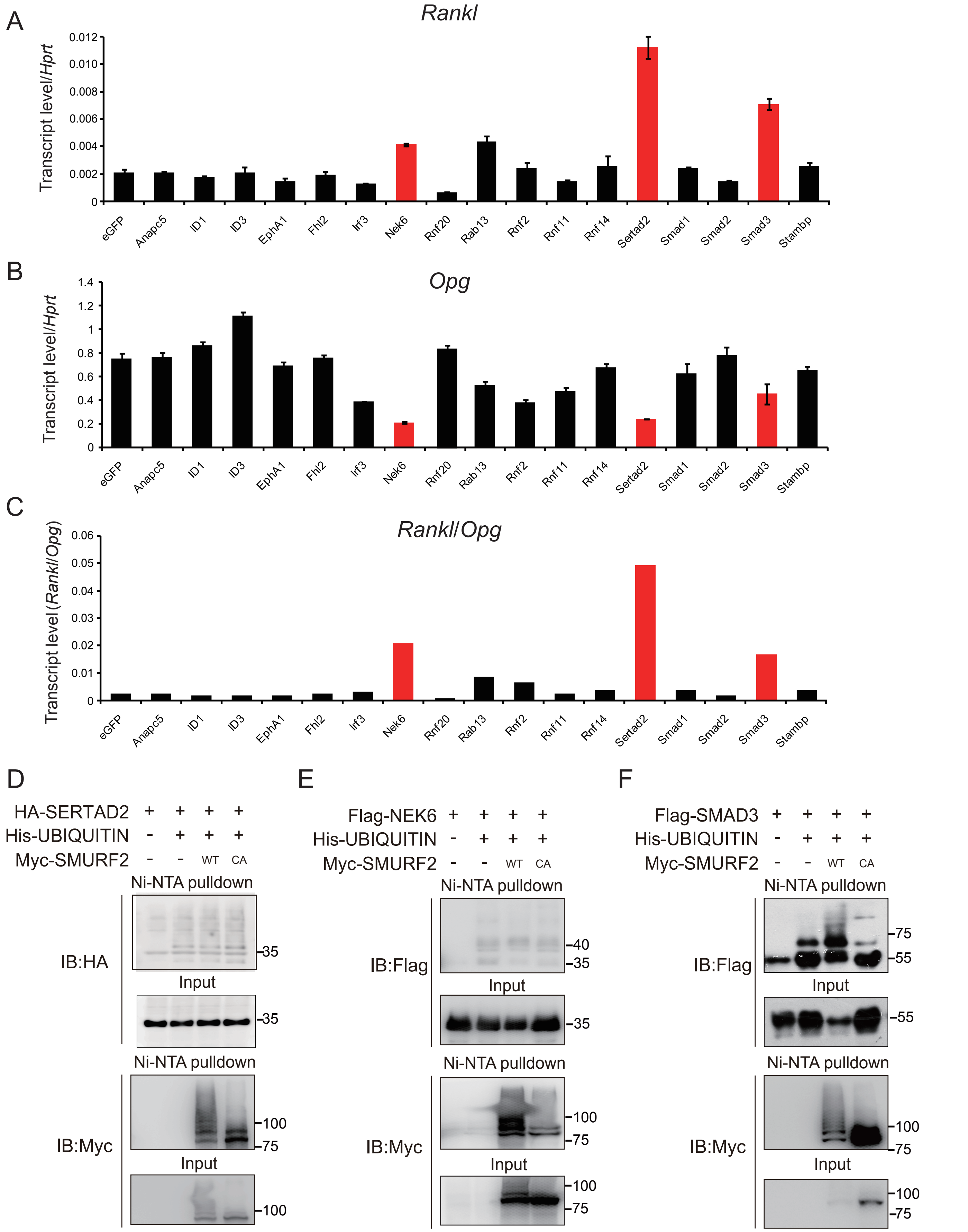

Supplementary Figure 3. The screen of the proteins known to interact with SMURF2. (A-C) mRNA expression of *Rankl*, *Opg* and the ratio of *Rankl*/*Opg* in C3H10T1/2 cells infected with lentivirus expressing the proteins known to interact with SMURF2. (D-F) HEK293T cells were transfected with the indicated plasmids, and cell lysates were precipitated with Ni-NTA beads and then immunoblotted with the indicated antibodies. All data are the mean±s.d.

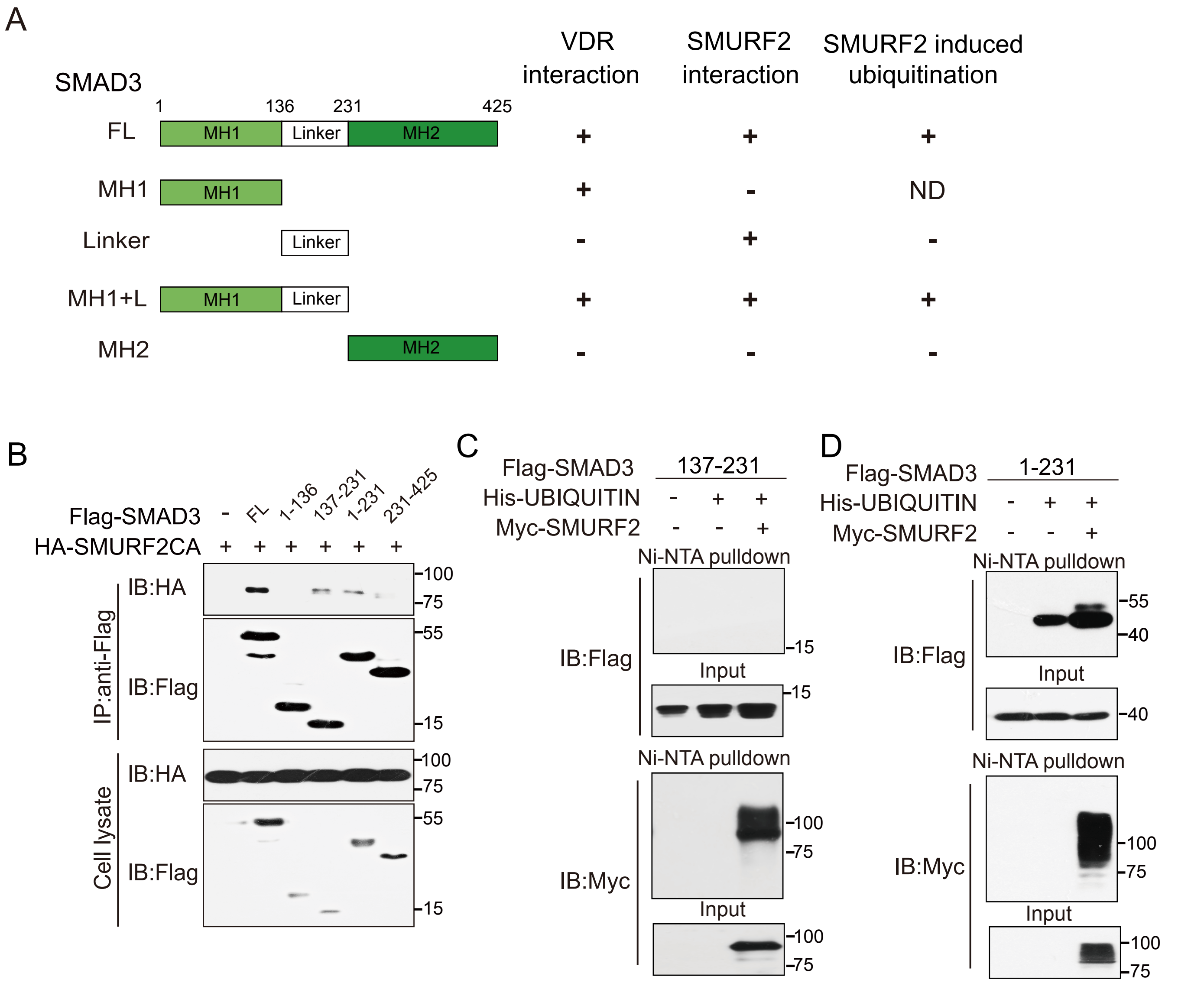

Supplementary Figure 4. Interaction of SMAD3 domains with VDR and SMURF2. (A) Interaction of SMAD3 domains with VDR and SMURF2. VDR and SMURF2 were tested for the interaction with the indicated truncations of SMAD3 in a GST–pull down assay and Co-immunoprecipitation assay (–, no interaction; +, interaction; ND, not detected). (B) Co-immunoprecipitation of SMAD3 truncations and VDR in 293T cells. (C-D) SMURF2 induced ubiquitination of the indicated truncations of SMAD3.

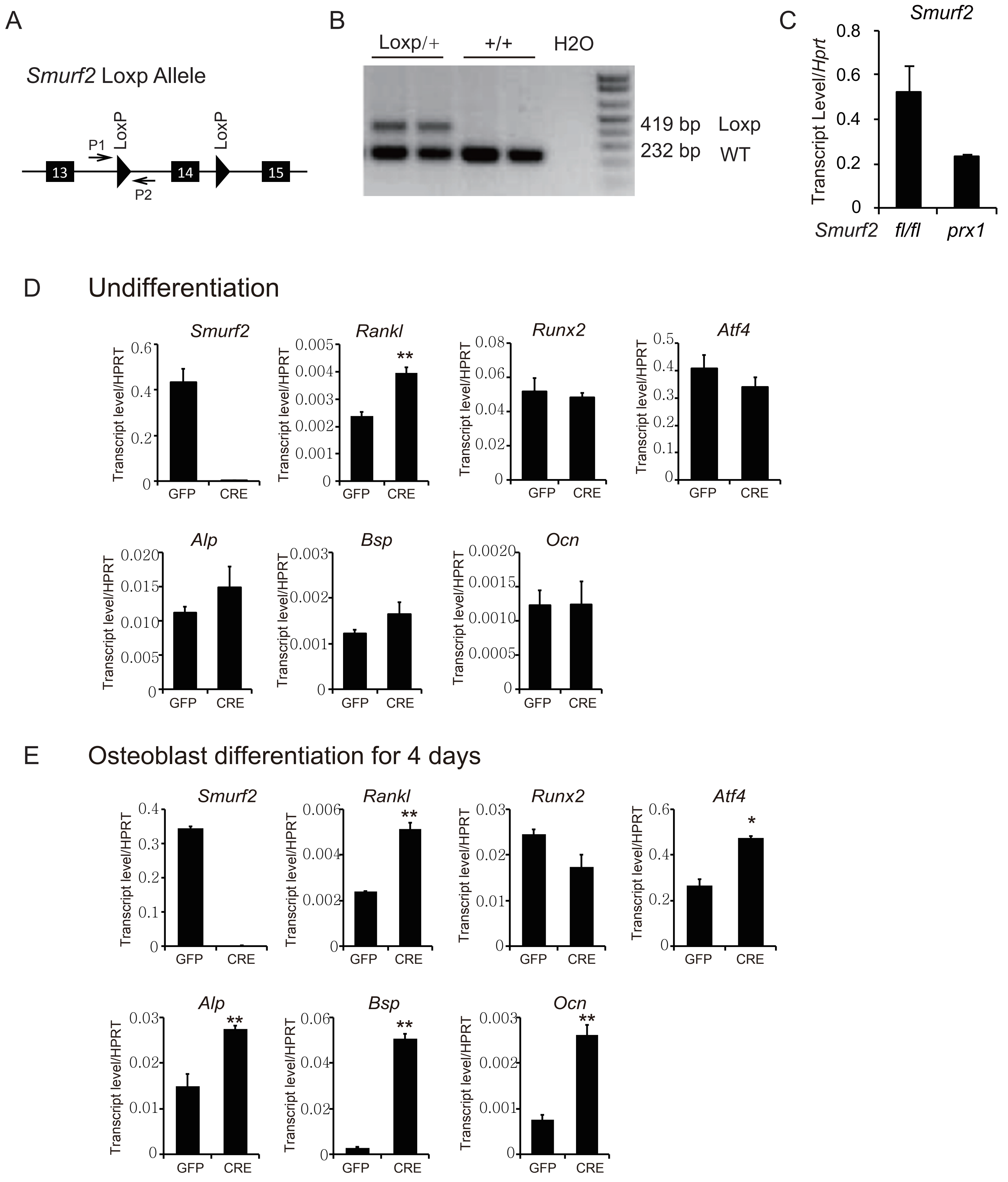

Supplementary Figure 5. Gene expression of the undifferentiated and differentiated Smurf2$^{F/F}$ osteoblasts infected by lentivirus expressing GFP and CRE. (A) Targeting strategy used to generate a floxed allele of *Smurf2*. (B) Genotype PCR analysis of WT and *Smurf2$^{fl/+}$* mice. (C) Expression of *Smurf2* mRNA from femur of *Smurf2$^{fl/fl}$* and *Smurf2^{prx1}* mice. (D) Analysis of gene expression of the undifferentiated Smurf2$^{F/F}$ osteoblasts infected by lentivirus expressing GFP and CRE. (E) Analysis of gene expression of Smurf2$^{F/F}$ osteoblasts infected by lentivirus expressing GFP and CRE cultured in osteoblast differentiation medium for 4d. All data are the mean±s.d. *P<0.05 , **P<0.01, ***P<0.001.Student's t-test was performed.

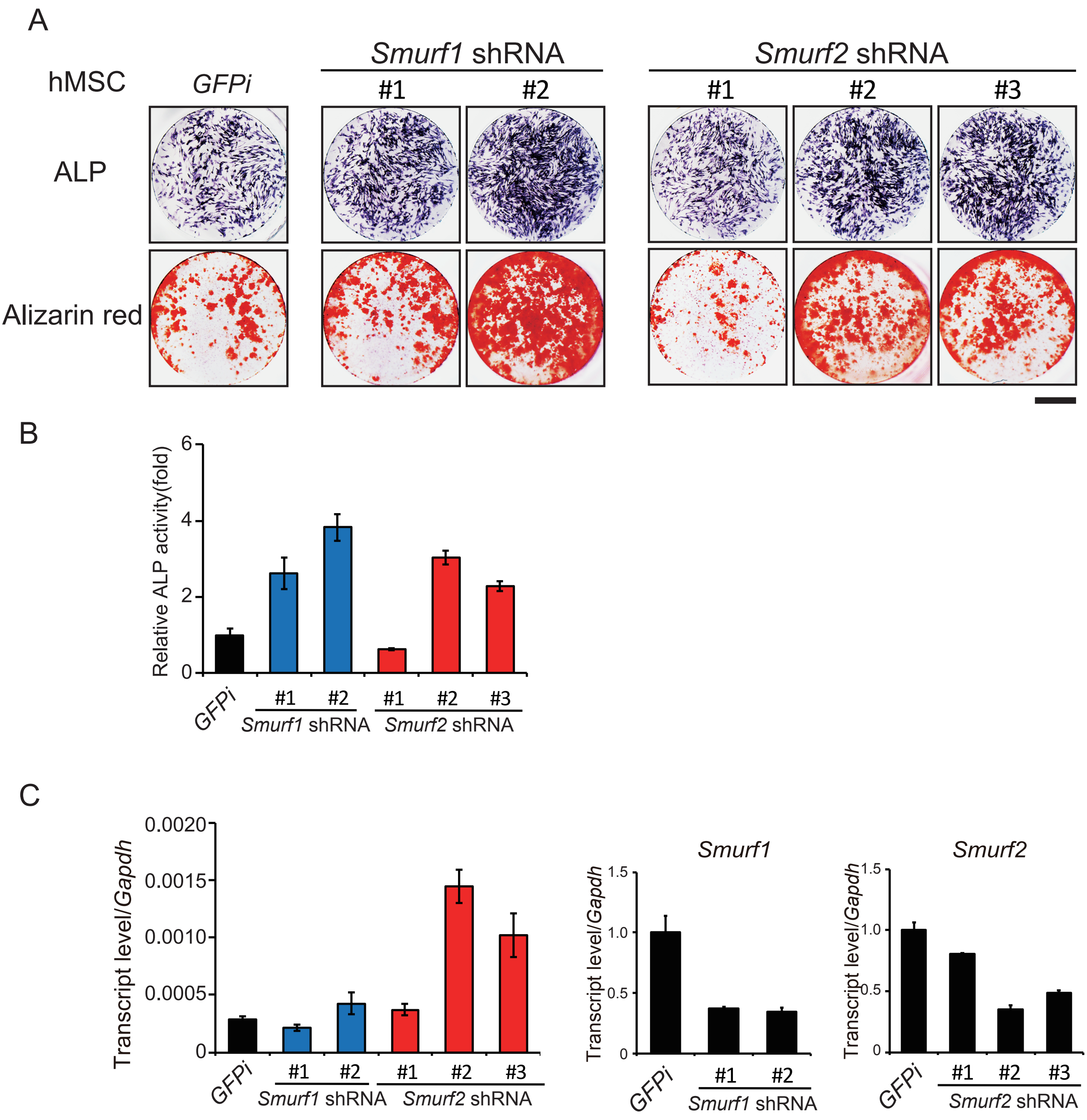

Supplementary Figure 6. The effects of SMURF2 and SMURF1 on the OB differentiation and *Rankl* expression with primary human mesenchymal stem cells. (A) Representative images for combined ALP(blue) and Alizarin red(red) staining of hMSCs infected with lentivirus expressing control or *Smurf1*/*Smurf2* shRNAs cultured in osteoblast differentiation medium for 7d and 14d, Scale bar, 0.25cm. (B) Analysis of ALP expression in hMSCs infected with lentivirus expressing control or *Smurf1*/*Smurf2* shRNAs, n=3 for each group. (C) qRT-PCR analysis of *Rankl* expression and knockdown efficiency of *Smurf1* and *Smurf2* in hMSCs infected with lentivirus expressing control or *Smurf1*/*Smurf2* shRNAs cultured in osteoblast differentiation medium for 7d.

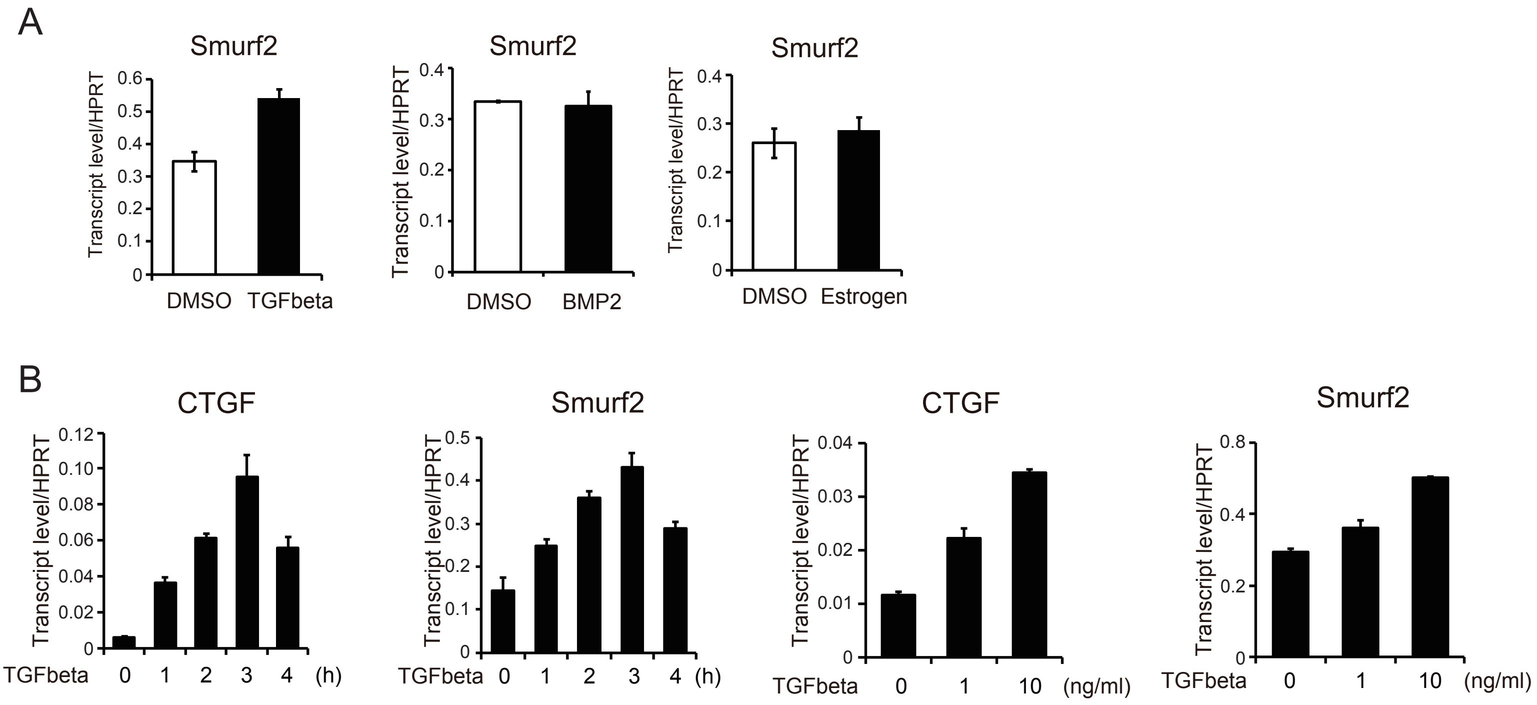

Supplementary Figure 7. TGF-β is the upstream regulator of Smurf2 expression. (A) Regulation of Smurf2 expression by potential upstream regulators. calvarial osteoblasts were cultured with different factors for 24h. TGF-β:10ng/ml; BMP2: 100ng/ml; Estrogen:100nM. (B) TGF-β could increase smurf2 expression in a dose-dependent and time-dependent manner. calvarial osteoblasts were cultured in the presence of 10ng/ml TGF-β for the indicated times or cultured with gradient concentrations of TGF-β for 3h, CTGF,as a potive control, is a downstream target gene of TGF-β signaling pathway .

Supplementary Figure 8

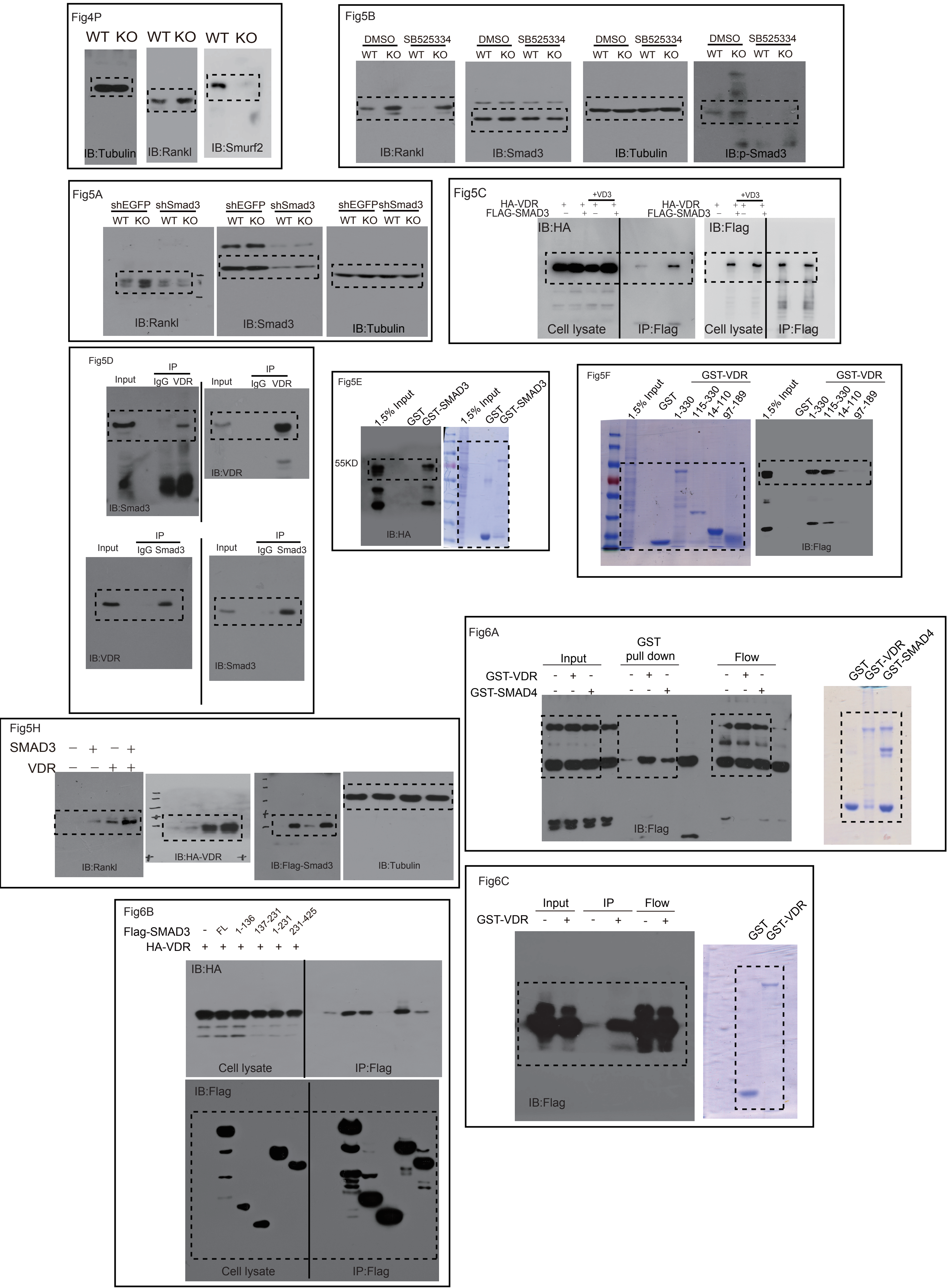

Supplementary Figure 8 - continued

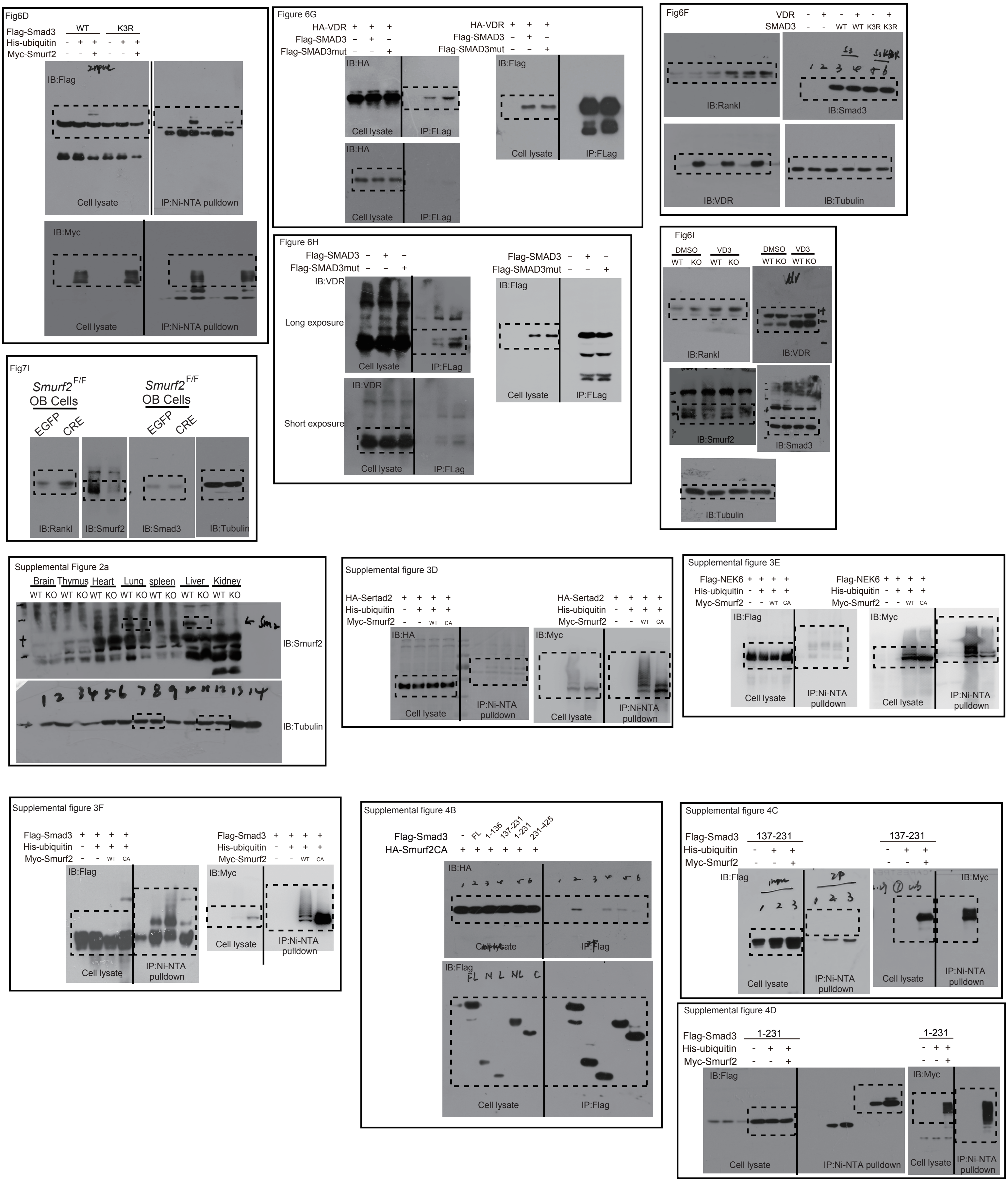

Parameters	WT (n=6)	*Smurf2* KO (n=6)	*p* value
BV/TV (%)	6.301±0.64	2.66±1.15*	0.022
Tb.Th (μm)	27.644±2.10	26.86±2.70	0.8287
Tb.N (/mm)	2.26±0.12	0.87±0.28**	0.0012
Tb.Sp (μm)	421±26	1607±381*	0.0112
MS/BS (%)	28.345±2.52	26.99±2.05	0.6986
MAR (μm/day)	2.35±0.11	2.21±0.17	0.5203
BFR/BS ($\mu m^3/\mu m^2$/year)	237±12	217±22	0.4514
BFR/BV (%/year)	1515±113	1565±149	0.8022
BFR/TV (%/year)	111±10	43±10***	0.0009
Ob.S/BS (%)	12.12±1.12	18.87±2.75*	0.0485
N.Ob/T.Ar (/mm^2)	48.088±3.82	25.72±6.89*	0.019
N.Ob/B.Pm (/mm)	10.83±1.14	15.58±1.32*	0.02461
OV/TV (%)	0.052±0.007	0.050±0.022	0.9268
OS/BS (%)	3.020±0.44	7.73±2.67	0.1135
O.Th (μm)	3.98±0.41	3.53±1.33	0.756
Oc.S/BS (%)	2.504±0.56	4.94±0.64*	0.0199
N.Oc/T.Ar (/mm^2)	3.788±1.04	3.76±1.79	0.989
N.Oc/B.Pm (/mm)	0.797±0.19	1.89±0.31*	0.015
ES/BS (%)	0.99±0.22	2.10±0.68	0.153

Supplementary Table 1. Histomorphometry analysis of *Smurf2* KO mice. 5 week old female *Smurf2* KO mice and wild type control mice were injected with calcein. 3 days later mice were injected with calcein again. 2 days later mice were sacrificed and tibias were processed for quantitative histomorphometric analysis at proximal tibiae, which revealed that *Smurf2* knockout mice had a significant decrease in cancellous bone volume (59%) and trabecular number (62%) with concomitant increase in trabecular spacing (281%) but no change in trabecular thickness, a characteristic of hyper-resorption phenotypes. Indeed, the decreased bone volume was associated with a significant increase in bone resorption parameters (Oc.S/BS and N.Oc/Bpm).

*p<0.05 compared to WT, unpaired t test

**p<0.01 compared to WT

****p*<0.001 compared to WT
